# TNF-α-Secreting Lung Tumor-Infiltrated Monocytes Play a Pivotal Role During Anti-PD-L1 Immunotherapy

**DOI:** 10.3389/fimmu.2022.811867

**Published:** 2022-04-14

**Authors:** Kirsten De Ridder, Hanne Locy, Elisa Piccioni, Miren Ibarra Zuazo, Robin Maximilian Awad, Stefaan Verhulst, Mathias Van Bulck, Yannick De Vlaeminck, Quentin Lecocq, Eva Reijmen, Wout De Mey, Lien De Beck, Thomas Ertveldt, Isabel Pintelon, Jean-Pierre Timmermans, David Escors, Marleen Keyaerts, Karine Breckpot, Cleo Goyvaerts

**Affiliations:** ^1^ Laboratory for Molecular and Cellular Therapy, Department of Biomedical Sciences, Vrije Universiteit Brussel (VUB), Brussels, Belgium; ^2^ Immunomodulation Group, Navarrabiomed, Navarrabiomed-UPNA-IdISNA, Pamplona, Spain; ^3^ Liver Cell Biology Research Group, Vrije Universiteit Brussel (VUB), Brussels, Belgium; ^4^ Laboratory of Molecular and Medical Oncology, Department of Biomedical Sciences, Vrije Universiteit Brussel (VUB), Brussels, Belgium; ^5^ Laboratory of Cell Biology & Histology, Antwerp Centre for Advanced Microscopy (ACAM), University of Antwerp, Antwerp, Belgium; ^6^ Rayne Institute, Division of Infection and Immunity, University College London, London, United Kingdom; ^7^ In Vivo Cellular and Molecular Imaging laboratory, Vrije Universiteit Brussel (VUB), Brussels, Belgium

**Keywords:** anti-PD-L1 immunotherapy, non small cell lung cancer (NSCLC), tumor-infiltrating myeloid cells, TNF-α, immunotherapy resistance

## Abstract

Immune checkpoint blockade (ICB) of the PD-1 pathway revolutionized the survival forecast for advanced non-small cell lung cancer (NSCLC). Yet, the majority of PD-L1^+^ NSCLC patients are refractory to anti-PD-L1 therapy. Recent observations indicate a pivotal role for the PD-L1^+^ tumor-infiltrating myeloid cells in therapy failure. As the latter comprise a heterogenous population in the lung tumor microenvironment, we applied an orthotopic Lewis Lung Carcinoma (LLC) model to evaluate 11 different tumor-residing myeloid subsets in response to anti-PD-L1 therapy. While we observed significantly reduced fractions of tumor-infiltrating MHC-II^low^ macrophages and monocytes, serological levels of TNF-α restored in lung tumor-bearing mice. Notably, we demonstrated *in vivo* and *in vitro* that anti-PD-L1 therapy mediated a monocyte-specific production of, and response to TNF-α, further accompanied by their significant upregulation of CD80, VISTA, LAG-3, SIRP-α and TIM-3. Nevertheless, co-blockade of PD-L1 and TNF-α did not reduce LLC tumor growth. A phenomenon that was partly explained by the observation that monocytes and TNF-α play a Janus-faced role in anti-PD-L1 therapy-mediated CTL stimulation. This was endorsed by the observation that monocytes appeared crucial to effectively boost T cell-mediated LLC killing *in vitro* upon combined PD-L1 with LAG-3 or SIRP-α blockade. Hence, this study enlightens the biomarker potential of lung tumor-infiltrated monocytes to define more effective ICB combination strategies.

## Introduction

Clinical blockade of the Programmed Death-1 (PD-1, CD279) pathway using monoclonal antibodies (mAbs) was added to the first-line treatment arsenal for advanced non-small cell lung cancer (NSCLC) patients in 2016 (1). This is justified by unprecedented tumor regression and long-term survival benefit in patients upon immune checkpoint blockade (ICB) of PD-1 or its ligand PD-L1 (CD274, B7-H1). However, most patients do not benefit from ICB, while responders often relapse or suffer from immune-related adverse events (2). This highlights the need for continued research in resistance mechanisms to identify additional druggable targets that can embellish the overall survival outcome for NSCLC patients to anti-PD-(L)1 therapies (3).

Preclinical studies suggest that PD-(L)1^+^ tumor-infiltrating myeloid cells, rather than tumor cells, determine the efficacy of ICB (4–12). In NSCLC, myeloid cells comprise a heterogeneous population of monocytic and granulocytic cells, including inflammatory, residential or Tie2^+^ angiogenic monocytes; alveolar next to monocyte-derived macrophages with divergent degrees of MHC-II expression; conventional type 1 and 2 dendritic cells (DCs); granulocytes like neutrophils and eosinophils, but also platelets and mast cells. Moreover, they have been shown to express PD-1 and PD-L1, vary in numbers and associate with tumor progression, immunosuppression, and therapy resistance (9, 13–17). Notably, anti-PD-L1 therapy has been shown to improve the immunostimulatory properties of particular myeloid cell subsets (18–21). Moreover, two recent studies reported that anti-PD-L1 therapy induces a systemic alteration of the monocytic compartment in NSCLC patients (22, 23). Nevertheless, how treatment with anti-PD-L1 mAbs affects the composition and function of different lung tumor-infiltrating myeloid subsets in an orthotopic NSCLC model has not been addressed so far.

In search for druggable targets to combine with ICB, we investigated the hypothesis that anti-PD-L1 therapy can induce myeloid cell-mediated resistance mechanisms to ICB therapy. Therefore, we mapped the impact of anti-PD-L1 therapy on 11 different lung tumor-infiltrating myeloid subsets in an ICB-unresponsive tumor model. More specifically we used an orthotopic Firefly luciferase (Fluc)-expressing squamous Lewis Lung Carcinoma (LLC) model to demonstrate that anti-PD-L1 therapy specifically decreases the fraction of MHC-II^low^ macrophages and monocytes within the lung tumor microenvironment. We further demonstrate that anti-PD-L1 therapy stimulates TNF-α secretion *in vivo* as well as *in vitro* with a prominent role, as source and sentinel, for the tumor-infiltrating monocytes. Moreover, the latter rendered more immunosuppressive monocytes demonstrated by elevated expression levels of the alternative checkpoint molecules VISTA, LAG-3, SIRP-α and TIM-3. Importantly, *in vitro* co-blockade of PD-L1 with LAG-3 or SIRP-α strongly boosted cytotoxic T cell (CTL)-mediated LLC killing. As the latter phenomenon was completely abolished in the absence of monocytes, this suggests a crucial role for monocytes in ICB combination effectiveness.

## Materials and Methods

### Mice, Cell Lines and Reagents

Six-week-old female C57BL/6J mice and transgenic OT-I mice were purchased from Charles River. All animals were handled according to the institutional guidelines, approved by the Ethical Committee Dierproeven (ECD) of the Vrije Universiteit Brussel for use of laboratory animals (ECD file numbers: 18-214-1, 18-281-9 and 20-214-12).

Lewis lung carcinoma (LLC) cells, kindly provided by Prof. Jo Van Ginderachter (VUB), were lentivirally transduced to express Firefly luciferase (LLC-Fluc) as described (24). For LLC-specific killing analysis, LLC cells were transduced with lentivectors encoding enhanced green fluorescent protein (eGFP) and ovalbumin (OVA), or Katushka (Kat) to generate LLC-eGFP/OVA or LLC-Kat, resp. Lentivector generation and cell line transductions are described in the **Supplementary Methods**. All LLC lines were maintained in DMEM^+^ i.e., Dulbecco’s-Modified-Eagle’s-Medium supplemented with 10% fetal bovine serum (FBS, TICO Europe), 100 units/mL penicillin, 100 µg/mL streptomycin and 2 mM L-Glutamine (all Sigma-Aldrich) at 37°C, 5% CO_2_, 21% O_2_ and 95% humidity level.

*In vivo* and *in vitro* recombinant mAb treatments were performed with rat IgG2b, K anti-PD-L1 mAb (clone: 10F9G2), rat IgG1, K anti-TNF-α mAb (clone: MP6-XT22); Ultra-LEAF Purified rat IgG1, K anti-mouse CD223 (LAG-3) mAb (clone: C9B7W), Ultra-LEAF Purified Armenian Hamster IgG anti-mouse PD-1H (VISTA) mAb (clone: MH5A), Ultra-LEAF Purified rat IgG1, K anti-mouse CD172a (SIRP-α) mAb (clone:P84), all from Biolegend next to InVivoMAb rat IgG2a, K anti-mouse TIM-3 mAb (clone: RMT3-23) from BioXcell. The according isotype controls were: Ultra-LEAF™ Purified rat IgG2b, K isotype control (Ctrl) mAb (clone: RTK4530) and rat IgG1, K Ctrl mAb (clone: RTK2071), both from Biolegend. All recombinant antibodies used to stain cells for flow cytometry are listed in **Supplementary Table 1**.

### Murine Tumor Cell Transfer, Treatment, and Monitoring

Mice were injected intravenously with 5x1e5 LLC-Fluc cells dissolved in 200 µl phosphate-buffered saline (PBS, Sigma-Aldrich). Control mice were challenged with 200 µl PBS only. Four days after LLC-Fluc challenge, mice were treated with four intraperitoneal injections of 200 µg anti-PD-L1 or rat IgG2b, K Ctrl mAb dissolved in 200 µl PBS given at 3-day intervals (days 4, 7, 10 and 13). To evaluate TNF-α blockade, 125 µg of anti-TNF-α or rat IgG1, K Ctrl mAb was administered on days 7, 10 and 13 instead. Tumor progression was monitored by body weight follow-up and *in vivo* bioluminescence imaging (BLI) as previously described (25). Briefly, mice were imaged for 7 min using the PhotonImager Optima (Biospace Lab), exactly 5 min after intravenous administration of 30 mg/kg substrate D-luciferin (Promega). Upon euthanasia, whole lungs were weighed and processed for whole-lung imaging or single-cell analysis.

### Preparation of Single-Cell Suspensions

Upon cervical dislocation, murine lungs were perfused with 5 ml PBS and transferred to 1 ml Roswell-Park-Memorial-Institute-1640 medium (RPMI-1640, Sigma-Aldrich) with 300 U/ml collagenase-I (Sigma-Aldrich). Lungs were chopped, incubated at 37°C for 45 min, and further mechanically reduced using a 18G syringe until single-cell suspensions could be passed through a 40 μm strainer. Spleens were transferred to 1 ml PBS, tamped, and passed through a 40 μm strainer. Blood (200 μl) was collected submandibularly into 1.3 ml LH microtubes (Sarstedt) and centrifuged for 10 min at 2000g to separate cell pellets from plasma. Murine femurs were isolated to flush out bone marrow with a 25G syringe in 1 ml PBS. Cell pellets were resuspended in 1 ml red blood cell lysis buffer for 5 min, followed by a wash step with PBS before analysis.

### Whole Lung Imaging

Lungs from 24 LLC tumor-bearing mice treated with Ctrl mAb and anti-TNF-α mAb or/and anti-PD-L1 mAb were isolated after perfusion with 5 ml PBS solution and fixed overnight at 4°C in 2 ml 0.05 M PBS containing 0.1 M L-lysine, 2 mg/ml NaIO4, and 10 mg/ml paraformaldehyde (PFA solution). The day after, lungs were rinsed in a 24-well plate (Costar) with PBS, consecutively immersed in a sucrose gradient (10%-20%-30%, 2 hrs at room temperature per gradient), embedded in optimal cutting temperature (OCT) compound and stored at -80°C. Frozen lungs were thawed in PFA solution and washed three times in PBS containing 0.5% Triton X-100 (washing buffer) at room temperature on a rotating shaker. For delipidation and antigenicity restoration after tissue fixation, lungs were submersed in FLASH buffer for overnight shaking (55 rpm) at 54°C, as described by Messal et al. (26). The following day, lungs were washed three times, be it with a first washing step in washing buffer with 1% H_2_O_2_ (Sigma). For nuclear staining, lungs were incubated with TO-PRO-3 (*Invitro*gen) diluted in ScaleCUBIC-1A buffer [as described by Susaki et al. (27)] at 37°C on a rotating shaker. Two days later, lungs were washed three times in PBS with 0.2% Tween 20 (Sigma) for 30 min on a rotating shaker. Subsequently, lungs were submersed in a 1:1 CUBIC-R: H_2_O solution for at least 6 hrs [as described by Susaki et al. (27)] and in 100% CUBIC-R solution for overnight incubation at room temperature. The following day, samples were embedded in a CUBIC-R 2% low melting agarose solution and fitted in to a 3D-printed mold (dimensions 2x1x0.8cm). For complete gelation, agarose-embedded lungs were kept at 4°C for at least 2.5 hrs after which they were immersed in CUBIC-R solution. For dehydration, samples were treated with a 1-propanol dilution series (25% - 50% - 75% - 100%) on a rotating shaker at room temperature for 30 min per dilution, and subsequently immersed in 100% 1-propanol for overnight shaking. For optical clearing, a similar concentration gradient was executed with ethyl cinnamate (ECi, Sigma) for 30 min each prior to their imaging using a Lavision Ultramicroscope II. Therefore, samples were immersed in ECi within a quartz cuvette and excited with light sheets of wavelengths 488 and 640 nm to record autofluorescent and TO-PRO-3 staining, resp. Image acquisition was performed on 2x magnification, 0.63x zoom and Z-stack steps of 10 µm. Files were saved as OME.TIF stacks and converted to Imaris files (.ims) using ImarisFileConverter (version 9.5.0), while tiles were stitched using ImarisStitcher (version 9.5.0). Three-dimensional images and tumor volumes were obtained with Imaris and quantified using surface creation algorithms.

### Flow Cytometry and Fluorescence-Activated Cell Sorting

Prior to staining, Fc-receptors were blocked using CD16/32 antibody (Biolegend) in cold FACS buffer i.e., PBS containing 1% bovine-serum-albumin and 0.02% sodium azide (Sigma-Aldrich). Next, staining was performed for 30 min at 4°C in FACS buffer. Fluorescently labelled cells were evaluated on a LSR Fortessa flow cytometer [Becton Dickinson (BD)], while analysis was performed using FlowJo_10.5.3 software. For myeloid subset sorts, the CD45^+^ immune cell fraction was enriched from whole lung tissue using anti-mouse CD45 MicroBeads (Miltenyi Biotec), prior to staining and FACS with a BD FACSAriaIII.

### LLC-Specific OT-I Killing Assay

Using the CD8α^+^ T-Cell-Isolation-Kit (Miltenyi Biotec), the CD8^+^ T-cell fraction was enriched from an OT-I mouse-derived splenic single-cell suspension. Next, 1e6 OT-I cells were resuspended in 1 ml RPMI-1640^+^ with 50 μmol/L β-mercaptoethanol and stimulated for two days with 20 μl Mouse T-Activator CD3/CD28 Dynabeads (Gibco). To evaluate target cell-specific killing, 6k LLC-eGFP/OVA cells were mixed with 6k LLC-Kat cells, 11k monocytes and 3k stimulated OT-I cells to obtain a target:effector ratio of 2:1 in 250 µl DMEM^+^. The added monocytes were either *in vitro* differentiated from bone marrow cells or isolated and MACS enriched using the Monocyte Isolation Kit and CD45 microbeads (Miltenyi Biotec, #130-100-629 and 130-052-301 resp.). While supernatants were collected 72hrs later for TNF-α and IFN-γ ELISA, cells were detached with 50 µl Trypsine-EDTA (Sigma-Aldrich) for 5 min at 37°C. LLC target cell-specific OT-I killing was evaluated by comparing the ratio of LLC-eGFP/OVA (target) over LLC-Kat (non-target) cells *via* flow cytometry. The percentage of specific killing normalized for the ratio in the cultures without OT-I cells was calculated with the following equation: 1−(%LLC-eGFP/OVA/%LLC-Kat) with CTLs/(%LLC-eGFP/OVA/%LLC-Kat) without CTLs. For spatiotemporal follow-up of target LLC-eGFP/OVA cell killing, eGFP intensity was monitored using Incucyte live cell imaging.

### Cytokine and Chemokine Detection

Serological concentrations of 31 cytokines and chemokines were measured using the Bio-Plex Pro_Mouse_Chemokine_Panel_31-Plex (#12009159, Bio-Rad). Samples were prepared and analyzed according to the manufacturers’ protocol using the Bioplex-200 instrument and Bio-Plex_Manager_6.0 software. Data points from standards that did not meet accuracy requirements were excluded prior to curve fitting. Standard curves obtained *via* 5PL logistic regression were used to determine the concentrations (pg/ml) for each analyte. Outliers were removed using the Interquartile Range method.

Protein levels of murine IFN-g and TNF-a were detected on supernatants collected from bone marrow-derived monocytes, splenocytes or the in vitro LLC-specific OT-I killing assay as described in the respective figure legends. Both ELISA kits (Invitrogen) were used according to the manufacturer’s protocol.

### Targeted Gene Expression Profiling

For each FACS-sorted lung-derived myeloid subset, 5x1e4 cells were collected in 500 μl Trizol and stored at -80°C. One to three weeks later, cell pellets were thawed, and RNA was isolated using the chloroform-isopropanol protocol. RNA recovery was quantified using fluorimetry (Qubit, LifeTechnologies) and qualified for purity and integrity using the nanophotometer (Implen) and Bioanalyzer Labchip (Agilent Technologies), resp. Due to low RNA concentrations, samples were pre-amplified using the nCounter Low-RNA-input kit (NanoString Technologies) with five amplification cycles. Sample hybridization was performed according to the manufacturers’ recommendations using the nCounter_Myeloid_Innate_ Immunity_Panel containing 770 genes. Hybridized samples were transferred to the nCounter Prep Station for further purification and immobilization to the sample cartridges. Absolute counts were quantified by the nCounter Digital Analyzer (nCounter MAX Analysis System, Brightcore, VUB). For quality control the nSolver_analysis_software_4.0 was used. Raw counts were processed in R and normalized using the RUVSeq method adjusted for NanoString analysis as described (28). Normalized counts were used to analyze differences between anti-PD-L1 and Ctrl mAb-treated samples. The DESeq R-package was used for differential expression analysis while gene-set-enrichment analysis (GSEA) was performed with settings: 1000 permutations, gene set selected as permutation type, and chip platform set to “Mouse_ENSEMBL_Gene_ID_Human_Orthologs_ MsigDB.v7.2”. For analysis, the Hallmark gene sets curated from the Molecular Signature Database (MSigDB) were used.

### Generation and Evaluation of Bone Marrow-Derived Monocytes

Primary bone marrow-derived cells from healthy female C57BL/6J mice, were plated at 4x1e6 in 10 ml DMEM^+^ supplemented with 20ng/ml macrophage colony-stimulating factor (M-CSF, Immunotools) in 100mmx15mm petri-dishes. Five days later, the non-adherent fraction was collected, representing a mixture of bone marrow-derived immature myeloid cells (**Figure 3C**). Next, 5x1e4 cells were plated per 200 µl DMEM^+^ in flat-bottom 96-well plates and subjected to 5 µg/ml anti-PD-L1 or Ctrl mAb and/or 10 ng/ml TNF-α (PeproTech). While supernatants were collected 24hrs later for TNF-α ELISA, monocytes were detached 72 hrs later using Accutase (Sigma-Aldrich) for 30 min at 37°C prior to flow cytometric analysis.

### Activation and Evaluation of Murine Splenocytes

Primary splenocytes from healthy female C57BL/6J mice were collected and plated at 2x10^5^ cells/200 µl RPMI-1640^+^ with 50 μmol/L β-mercaptoethanol and 30 ng/ml IL-2 (Peprotech) per well of a U-bottom 96-well plate. After overnight stimulation with 20 μl/10^6^ cells of Mouse T-Activator CD3/CD28 Dynabeads (Gibco), cells were treated for 72hrs with 1 µg/ml recombinant TNF-α (PeproTech), with or without 10 µg/ml Ctrl mAb or anti-PD-L1 mAb after which supernatants were collected for IFN-γ ELISA.

### Statistics

The asterisk number in the figures indicates the level of statistical significance as follows: * for p < 0.05; ** for p < 0.01; *** for p < 0.001 and **** for p < 0.0001. The statistical test used to determine statistical significance is indicated in the figure legends. Statistical tests were performed using RStudio v1.3.1056 or Graphpad Prism v8.3.0. Heatmaps were constructed with the heatmap package in R. Serological concentrations are scaled by subtracting the mean and dividing by standard deviation for each sample. For GSEA, gene sets were identified as significant when nominal (NOM) p-value < 0.01, false discovery rate (FDR) < 0.25, and normalized enrichment score (NES) > 1.

## Results

### Anti-PD-L1 Therapy Has No Therapeutic Benefit Despite PD-(L)1 Expression

**Figure 1:**
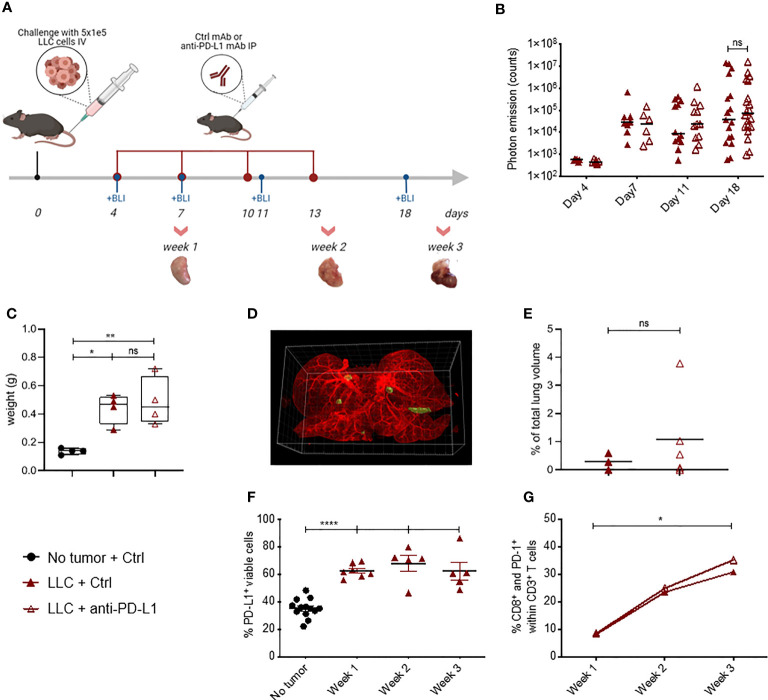
Blockade of PD-L1 does not delay LLC-Fluc tumor progression, despite PD-(L)1 expression. **(A)** Schematic representation of the experimental set-up. Mice were challenged intravenously (IV) with LLC-Fluc cells and treated every 3 days with anti-PD-L1 or isotype control (Ctrl) mAbs for 4 times in total (red dots). LLC-Fluc engraftment was monitored using *in vivo* bioluminescence imaging (BLI) on the indicated time points (blue dots), while on weeks 1, 2 and 3, lungs were also isolated for flow cytometric evaluation. **(B)** Summary of photon counts collected upon BLI measurements. On a logarithmic scale, the absolute number of counts per analyzed day (on x-axis) are shown (n=3, 5 mice per condition [mpc]). A two-way ANOVA with Sidak’s multiple comparisons test was performed to evaluate statistical significance. **(C)** Weights of whole lungs, isolated from healthy or tumor-bearing mice sacrificed three weeks after cancer initiation (n=1, 4-5 mpc). **(D)** A representative 3D image that is used for volumetric quantification of tumor volumes (green) in an Ctrl mAb-treated murine lung (autofluorescence, red), obtained with Imaris software. **(E)** Percentage of tumor volume over whole lung volume to compare LLC engraftment in mice treated with Ctrl mAbs or anti-PD-L1 mAbs (n=3-4). Statistical significance was measured using an unpaired t-test. **(F)** Graph representing the percentages of PD-L1^+^ cells found upon flow cytometry analysis of the viable lung single-cell fraction, isolated from healthy or LLC-Fluc tumor-bearing mice on weeks 1-3 after tumor challenge (n=3, 1-3 mpc). **(G)** Percentage of lung tumor-infiltrated PD-1^+^ CD8^+^ T-cells within the CD3^+^ T-cell population at 1, 2 and 3 weeks after LLC-Fluc inoculation analyzed *via* flow cytometry (n=3, 1-3 mpc). A two-way ANOVA with Tukey’s multiple comparisons test was performed to determine statistical significance in panels **(C, F, G)**. ns, non-significant; * for p < 0.05; ** for p < 0.01; and **** for p < 0.0001.

Lewis Lung Carcinoma (LLC) represents a highly tumorigenic mesenchymal K-Ras mutant squamous lung cancer model, characterized by low tumor cell-specific PD-L1 expression with limited response to anti-PD-L1 therapy (5, 6). As Fluc^+^ tumor lines have been shown to be more immunogenic (30), we first evaluated if LLC-Fluc tumors were similarly unresponsive to treatment with anti-PD-L1 or isotype control (Ctrl) mAbs (**Figure 1A**). Follow-up of body weights and tumor load (bioluminescent signals) over time demonstrated that anti-PD-L1 therapy does not reduce tumor progression (**Supplementary Figures 1A, B** resp.). Three weeks after engraftment, mice were sacrificed to isolate whole lungs. Similar lung weights (**Figures 1C**), tumor foci volumes (**Figures 1D, E**), numbers and average volumes (**Supplementary Figures 1B, C**), further confirmed the lack of therapeutic effect upon anti-PD-L1 therapy.

Anti-PD-L1 therapy can only lead to stimulation of antitumor immunity if the PD-1 pathway is exploited. Therefore, we evaluated whether absence of PD-1 and/or PD-L1 in the lung tumor microenvironment could account for therapy failure in the orthotopic LLC-Fluc model. Flow cytometric analysis of lungs, isolated 1, 2 and 3 weeks after LLC-Fluc challenge, showed that the overall percentage of PD-L1^+^ pneumocytes increased during LLC-Fluc progression compared to healthy controls (**Figure 1F** with gating strategy in **Supplementary Figure 1D**). Also *in vitro*, flow cytometric analysis of LLC-Fluc cells endorsed their ability to express and upregulate PD-L1 upon exposure to IFN-γ (**Supplementary Figure 1E**). Moreover, we observed a significant increase in the percentage of PD1^-+^ CD8^+^ T-cells over time irrespective of anti-PD-L1 therapy (**Figure 1G** with gating strategy in **Supplementary Figure 2A**). In parallel, while CD4^+^ T- and B-cell fractions decreased upon tumor progression, their respective PD-1^+^ portions also increased significantly (**Supplementary Figures 2B–E**). To conclude, despite significant PD-1 and PD-L1 expression in the orthotopic LLC-Fluc model, anti-PD-L1 therapy does not delay tumor progression.

### LLC Progression Is Accompanied by a Local and Systemic Increase in Monocytes

**Figure 2:**
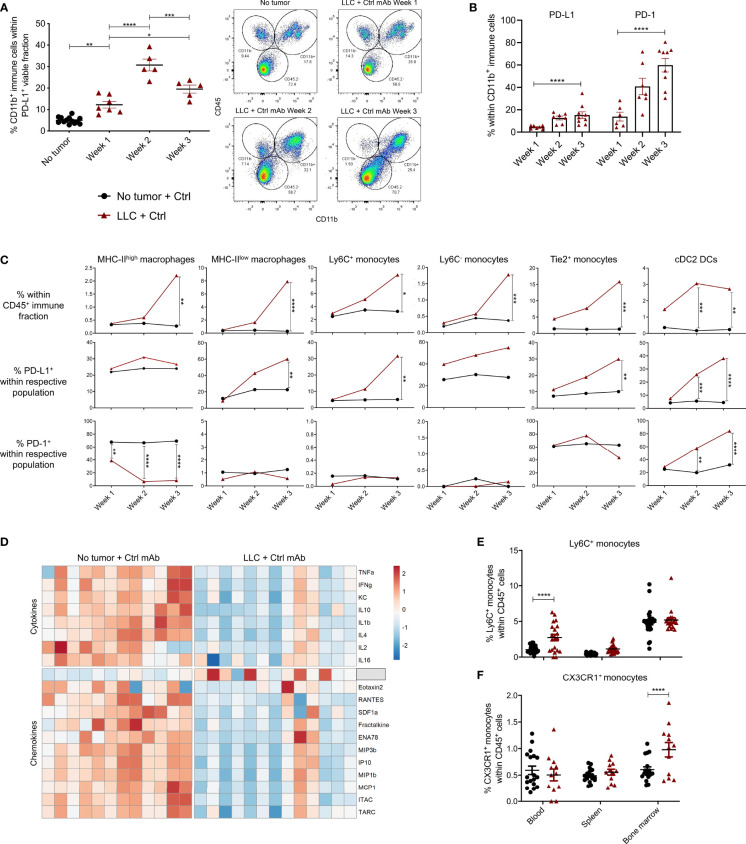
PD-L1^+^ and PD-1^+^ myeloid cells in healthy and LLC-engrafted mice. Lungs of healthy and LLC-Fluc tumor-bearing mice were isolated on weeks 1, 2 and 3 after LLC-Fluc challenge to evaluate PD-L1 and PD-1 expression in flow cytometry. **(A)** Summary of percentages of CD45^+^ CD11b^+^ myeloid cells within the viable PD-L1^+^ fraction (left panel) with representative dot plots (right panel, n=3, 1-3 mpc). **(B)** Evolution of myeloid cell-specific PD-L1 and PD-1 expression over time in lungs from LLC-bearing mice (n=3, 1-3 mpc). **(C)** Follow-up of MHC-II^high^ or MHC-II^low^ F4/80^+^ macrophages; Ly6C^+^, Ly6C^-^ or Tie2^+^ monocytes and CD11b^+^ CD103^-^ cDC2 within the CD45^+^ immune fraction from healthy (no tumor) or LLC-engrafted lung tissue over the course of 3 weeks (upper row), next to their respective expression of PD-L1 (middle row) and PD-1 (lower row) (n=3, 1-3 mpc). **(D)** Heatmap of significantly altered cytokines and chemokines in serum of mice, collected 3 weeks after challenge with LLC-Fluc or PBS only. Per chemo-/cytokine, concentrations were centered and scaled resulting in a color scale ranging from blue to red for lowest to highest concentration values. Statistics were performed using one-way ANOVA followed by Tukey’s multiple comparison test (F-value= 8.97, Df= 2) (n=2, 4-8 mpc). **(E, F)** Percentage of Ly6C^+^ **(E)** and CX3CR1^+^ **(F)** monocytes, within viable CD45^+^ single-cells from blood, spleen and bone marrow collected from mice 3 weeks after inoculation with PBS or LLC-Fluc cells (n=3, 5-10 mpc). For panels **(A–C, E, F)** a two-way ANOVA with Sidak’s multiple comparisons test was performed to evaluate statistical significance. ns, non-significant; * for p < 0.05; ** for p < 0.01; *** for p < 0.001; and **** for p < 0.0001.

In light of add-on therapies that can improve the response to anti-PD-L1 therapy, it is crucial to understand if, which and how PD-L1^+^ cells respond *in vivo*. Therefore, we first deciphered which cells were PD-L1^+^ and thus could act as potential targets for anti-PD-L1 therapy. When the portions of CD45^-^ (non-immune), CD45^+^ (immune) or/and CD11b^+^ (myeloid) cells were determined within the total PD-L1^+^ fraction, a significant increase of myeloid cells was shown from week 1 onwards after LLC-Fluc challenge (**Figure 2A**). In accordance with recent reports on the negative impact of PD-1^+^ myeloid cells on T-cell amplification responses (31, 32), PD-1 positivity was also evaluated, showing a similar five-fold increase for PD-1 and PD-L1 within the tumor-infiltrated CD11b^+^ fraction upon LLC-Fluc progression (**Figure 2B**). Next, we assessed changes in abundance and PD-(L)1 expression level of 11 different myeloid subsets (gating strategy in **Supplementary Figure 3A** *)*. Upon tumor progression, 6 lung-residing myeloid subsets increased compared to healthy mice: MHC-II^high^ and MHC-II^low^ tumor-associated macrophages (TAMs), inflammatory Ly6C^+^ monocytes, residential Ly6C^-^ monocytes and angiogenic Tie2^+^ monocytes next to conventional type 2 dendritic cells (cDC2) (**Figure 2C**, upper row). Moreover, these subsets increased their PD-L1 expression over time, except for the MHC-II^high^ TAMs (**Figure 2C**, middle row). Further, cDC2s increased their PD-1 expression while the opposite was found for MHC-II^high^ TAMs (**Figure 2C**, lower row). In contrast, the fraction of alveolar macrophages and eosinophils decreased significantly during tumor progression, while mast cells, cDC1 and neutrophil portions remained status quo (**Supplementary Figure 3B**). The systemic impact of LLC-Fluc engraftment was evaluated by serological evaluation of 31 chemo- and cytokines next to determination of the myeloid composition in blood, spleen and bone marrow. In accordance with the increase in TAMs and monocytes linked to LLC-Fluc progression, macrophage chemoattracting protein‐3 (MCP-3 or CCL7) circulated at a significantly higher level in LLC-bearing animals (**Figure 2D** and **Supplementary Table 2**). In contrast, the serological level of IFN-γ was significantly decreased in LLC-bearing mice compared to healthy controls. Finally, the lung-specific increase in monocytes (but not of TAMs nor cDC2s – data not shown), was reflected systemically by their significant rise in blood (**Figure 2E**) and bone marrow resp. (**Figure 2F**).

### Anti-PD-L1 Therapy Abolishes the Rise in MHC-II^low^ TAMs and Monocytes Upon LLC Progression

**Figure 3:**
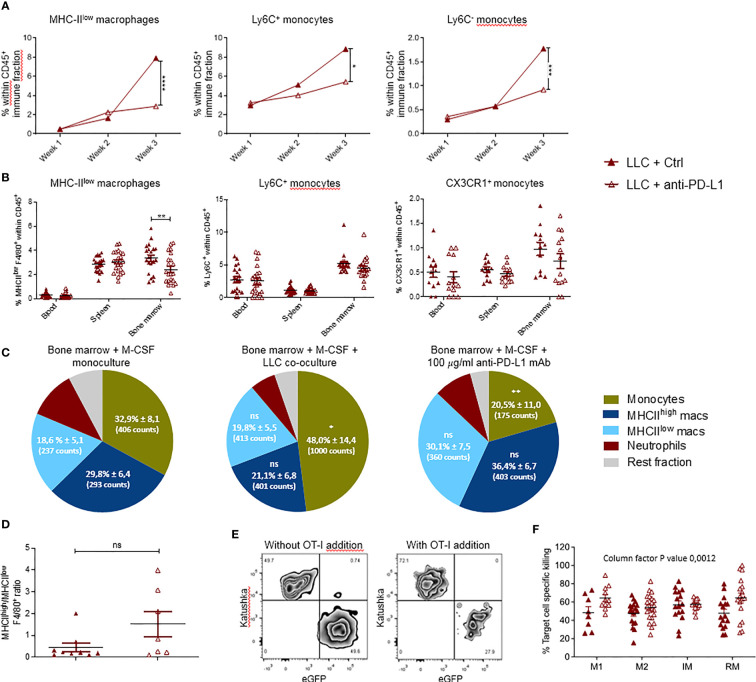
Local and systemic impact of anti-PD-L1 therapy on myeloid cells. Mice were treated with Ctrl or anti-PD-L1 mAbs on days 4, 7, 10 and 13 after LLC-Fluc injection. On weeks 1, 2 and 3, mice of each group were sacrificed to isolate LLC-engrafted lungs. Blood, spleen and bone marrow were collected on week 3. **(A)** Graphs indicating changes in percentage of lung-infiltrated MHC-II^low^ macrophages (left), Ly6C^+^ monocytes (middle) and Ly6C^-^ monocytes (right) within the CD45^+^ immune cell fraction assessed using flow cytometry (n=3, 1-3 mpc). **(B)** Percentages of MHC-II^low^ macrophages (left), Ly6C^+^ monocytes (middle) and CX3CR1^+^ monocytes (right) in peripheral organs as depicted on the x-axis (n=3, mpc=5-10). Two-way ANOVA with Sidak’s multiple comparisons test was performed to evaluate statistical significance. **(C)** Bone marrow-derived cells were cultivated with monocyte colony-stimulating factor (M-CSF) for 5 days. Next, cells were left untreated (left), co-cultured with LLC cells (middle) or 100µg/ml anti-PD-L1 mAb (right). One day later, alterations in myeloid subset composition were evaluated *via* flow cytometry. Percentages (± sd) from the total CD11b^+^ population are shown. Percentages are accompanied by the average counts for the surface markers MHC-II^low^, MHC-II^high^ and Ly6C to describe the mean fluorescence intensity of MHC-II^low^ macrophages, MHC-II^high^ macrophages and monocytes resp. (n=5). **(D)** Graphical representation of the altered ratio of lung-residing MHC-II^high^ over MHC-II^low^ TAMs isolated from tumor-bearing mice treated with Ctrl or anti-PD-L1 mAbs. An unpaired t-test was performed to analyze statistical significance (n=3, 2-3 mpc). **(E)** Representative zebra plot of the LLC-specific OT-I killing assay with gating of target LLC-eGFP/OVA and non-target LLC-Kat cells, with or without pre-stimulated CD8^+^ OT-I T cells. **(F)** Percentage target cell-specific killing in the presence of MHC-II^high^ TAM, MHC-II^low^ TAM, Ly6C^+^ inflammatory (IM) and CX3CR1^+^ residential (RM) monocytes sorted from lung tumor-bearing mice, treated with Ctrl or anti-PD-L1 mAbs (n=5, ≧3 biological replicates). For determination of statistical analysis in **(A, C, F)**, a two-way ANOVA with Tukey’s multiple comparisons test was performed. ns, non-significant; * for p < 0.05; ** for p < 0.01; *** for p < 0.001; and **** for p < 0.0001.

The impact of anti-PD-L1 therapy on the local and peripheral myeloid cell distribution in LLC-bearing animals was evaluated using the experimental design shown in **Figure 1A**. Briefly, LLC-bearing mice were treated with Ctrl or anti-PD-L1 mAbs given 4 times at 3-day intervals. Lungs were isolated on weeks 1, 2 and 3 to evaluate the dynamics of 11 different lung-residing myeloid subsets. No significant differences were found for neutrophils, eosinophils, mast cells, alveolar macrophages, MHC-II^high^ TAMs, cDC1s or cDC2s (**Supplementary Figure 4**). In contrast, the elevation in MHC-II^low^ TAMs and monocytes linked to LLC progression was abolished by anti-PD-L1 therapy (**Figure 3A**). Also, anti-PD-L1 therapy resulted in a significantly reduced fraction of MHC-II^low^ macrophages residing in the bone marrow next to an insignificant trend towards reduced numbers of inflammatory and patrolling monocytes in blood and bone marrow (**Figure 3B**). To understand if this reduction was linked to a direct impact of anti-PD-L1 mAbs on macrophages and/or monocytes, we treated primary bone marrow-derived myeloid cells with anti-PD-L1 or Ctrl mAbs. In line with the *in vivo* data, co-cultivation with LLC cells resulted in a significant increase of the MHC-II^low^ Ly6C^+^ monocyte fraction *in vitro*, while anti-PD-L1 mAb-treatment resulted in a significant decrease compared to the untreated monoculture (**Figure 3C**). In contrast, no significant changes were observed within the F4/80^+^ macrophage populations. We further evaluated the ratio of MHC-II^high^ over MHC-II^low^ TAMs *in vivo* and only found an insignificant trend towards more MHC-II^high^ TAMs suggesting that anti-PD-L1 therapy in the LLC model was not able to install a pronounced immunostimulating environment (**Figure 3D**). Finally, we sorted the lung tumor-infiltrated MHC-II^high^ and MHC-II^low^ TAMs and Ly6C^+^ inflammatory and CX3CR1^+^ residential monocytes from 3-week LLC-engrafted mice to evaluate their capacity to boost LLC target cell-specific killing *ex vivo* (**Figures 3E, F**). We observed that anti-PD-L1 therapy only modestly improved the capacity of the MHC-II^high^ TAMs and residential monocytes to boost CTL-mediated LLC eradication.

### Anti-PD-L1 Therapy Results in a Monocyte-Specific TNF-α Response

**Figure 4:**
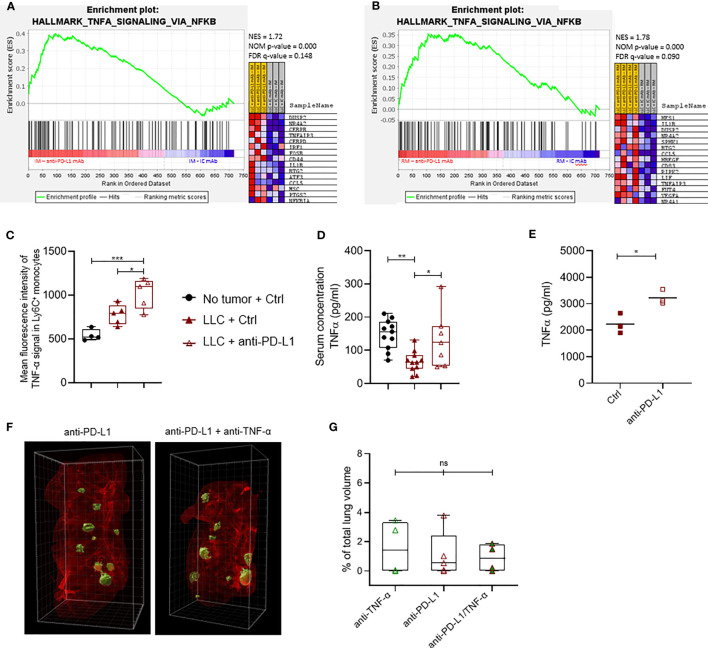
Anti-PD-L1 therapy induces a TNF-α-linked response in monocytes. Three weeks after LLC engraftment, lungs from anti-PD-L1 or Ctrl mAb-treated mice were isolated and pooled to sort minimally 50k Ly6C^+^ and CX3CR1^+^ monocytes. Subsequently, RNA was isolated to perform targeted gene expression profiling using the Nanostring platform. **(A, B)** Gene set enrichment analysis ranking genes using the MSigDB Hallmark gene set collection for Ly6C^+^ and CX3CR1^+^ monocytes resp. with NES, NOM p-value and FDR q-value denoted on the graphs. On the right, the top 15 gene set members on the rank ordered list are depicted (n=1, mpc=3-4). **(C)** Mean fluorescence intensity of cytoplasmic TNF-α measured *via* flow cytometry in Ly6C^+^ monocytes isolated from healthy, or LLC-bearing mice treated with Ctrl or anti-PD-L1 mAb (n=1, 4-5 mpc). **(D)** Boxplots representing the serological concentration of TNF-α in blood of healthy or LLC-bearing mice treated with Ctrl or anti-PD-L1 mAb resp. (n=2, mpc=4-8). **(E)** Dot plot depicts the concentration of TNF-α measured with ELISA in the supernatant of *in vitro* generated bone marrow-derived monocytes, treated with Ctrl or anti-PD-L1 mAbs for 24hrs. Statistical significance was measured with an unpaired t-test (n=3, 3 technical replicates). **(F)** Four days after LLC-Fluc inoculation, mice were treated with anti-PD-L1 mAb. On days 7, 10 and 13, mice received consistently one of the following 3 treatments: Ctrl + anti-TNF-α mAb; Ctrl + anti-PD-L1 mAb or anti-PD-L1 + anti-TNF-α mAb. Three weeks later, mice were euthanized, and perfused lungs were fixed for whole-tissue imaging. Representative 3D images obtained with lightsheet microscopy show the volumetric tumor foci (green) in lung tissue (red) of anti-PD-L1 mAb alone or anti-TNF-α + anti-PD-L1 mAb-treated mice. **(G)** Percentage of tumor volume over whole lung volume to compare LLC engraftment in mice treated with anti-TNF-α mAbs, anti-PD-L1 mAbs or a combination thereof (mpc = 4). Statistical significance in panels **(C, D, F)** were determined with a one-way ANOVA followed by a Tukey’s multiple comparisons test. ns, non-significant; * for p < 0.05; ** for p < 0.01; and *** for p < 0.001.

As anti-PD-L1 therapy does not result in LLC growth reduction while it modestly increases the immunostimulatory profile of TAMs and monocytes, we used the nCounter Myeloid_Innate_Immunity_Panel to uncover more subtle myeloid subset-specific transcriptional changes upon anti-PD-L1 therapy *in vivo*. While no significant differentially expressed genes were found within sorted CX3CR1^+^ monocytes or MHC-II^low^ TAMs, *Dusp2* and *Stab1* were significantly up-and down-regulated resp., in inflammatory monocytes derived from anti-PD-L1 mAb-treated mice (**Supplementary Figure 5A**). Using Gene Set Enrichment Analysis (GSEA) with the hallmark gene-set collection of MSigDB, we found that MHC-II^low^ TAMs from anti-PD-L1-treated mice significantly decreased their expression of an IFN-γ response linked gene set, again suggesting that anti-PD-L1 therapy does not support stimulation of an IFN-γ-linked CTL response *in vivo* (**Supplementary Figure 5B**). In contrast, monocytes of anti-PD-L1-treated mice showed significant enrichment for a gene set that is regulated by NF-kB in response to TNF-α (**Figures 4A, B**). Accordingly, these monocytes showed a significantly increased level of intracytoplasmic TNF-α compared to healthy LLC-bearing Ctrl mAb-treated mice (**Figure 4C**). In contrast, we did not observe this TNF-α increase in the MHC-II^high^ nor ^low^ macrophages upon anti-PD-L1 therapy (**Supplementary Figures 5C, D** resp.). In congruence, anti-PD-L1 mAb treatment rectified the serological drop of TNF-α caused by LLC engraftment (**Figure 4D**). When bone marrow-derived immature myeloid cells were treated with anti-PD-L1 mAbs *in vitro*, we confirmed significantly elevated levels of TNF-α in these supernatants compared to Ctrl mAb treatment (**Figure 4E**). Encouraged by these data and previous reports on TNF-α blockade to improve anti-PD-1 therapy efficacy in a mouse model of melanoma and colon carcinoma (33, 34), we evaluated the therapeutic potential of TNF-α and PD-L1 co-blockade in the LLC-Fluc model. Mice were treated with anti-PD-L1 mAbs on day 3 after LLC-Fluc cell injection and subsequently on days 7, 10 and 13 with anti-TNF-α mAbs, anti-PD-L1 mAbs or a combination thereof. Upon 3D whole lung tissue imaging 3 weeks later, no significant differences were found in terms of total tumor volume, number of foci or average foci volume (**Figures 4F, G** and **Supplementary Figures 5E, F**), showing no therapeutic benefit of TNF-α blockade in the anti-PD-L1-treated orthotopic LLC model.

### TNF-α Fortifies the Upregulation of Checkpoint Molecules on Anti-PD-L1-Treated Monocytes

**Figure 5:**
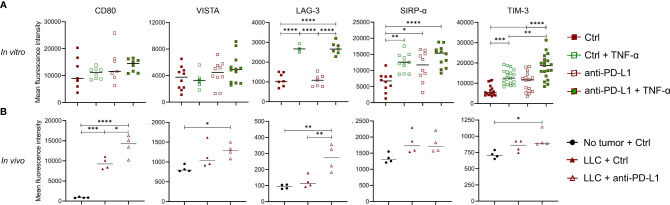
Anti-PD-L1 therapy upregulates checkpoints on monocytes. **(A)** Bone marrow-derived monocytes were plated with LLC cells and treated with 5 µg/ml Ctrl or anti-PD-L1 mAb, with or without 10 ng/ml TNF-α. Three days later, mean fluorescence intensities were obtained *via* flow cytometry for CD80, VISTA, LAG-3, SIRP-α and TIM-3 (n=3, 4 technical replicates). **(B)** Three weeks after LLC engraftment, lungs from anti-PD-L1 or Ctrl mAb-treated mice were isolated to enrich the Ly6C^+^ monocytes and evaluate their expression of the same checkpoints as described in panel **(A)** As internal reference, monocytes derived from healthy mice were taken along. A two-way panel **(A)** or one-way panel **(B)** ANOVA with Tukey’s multiple comparisons test was performed to determine statistical significance. * for p < 0.05; ** for p < 0.01; *** for p < 0.001; and **** for p < 0.0001.

Although previous reports have shown that TNF-α blockade can elevate ICB effectiveness, we were unable to confirm this in the orthotopic LLC model. Since anti-PD-L1 therapy induces the secretion of TNF-α in monocytes *in vitro* and *in vivo*, we wondered what their combined immunological effect was on monocytes. Therefore, we treated *in vitro* generated bone marrow-derived monocytes with anti-PD-L1 mAbs and/or recombinant TNF-α. After 72 hrs, Ly6C^+^ monocytes were analysed for their expression of the immunostimulatory checkpoints CD80 (B7.1) and Inducible-T-Cell-Costimulator-Ligand (ICOSL) next to the immunosuppressive checkpoints: V-domain-Ig-suppressor-of-T-cell-activation (VISTA), Lymphocyte-Activating-3 (LAG-3), signal-regulatory-protein-α (SIRP-α), T-cell-immunoglobulin-and-mucin-domain-containing-3 (TIM-3), carcinoembryonic-antigen-related-cell-adhesion-molecule-1 (Ceacam1), T-Cell-Immuno-receptor-Ig-and-ITIM-domains (TIGIT), CD276 (B7-H3) and CD73. Flow cytometric analysis showed no alterations for ICOSL, Ceacam1, TIGIT, CD276 nor CD73 (data not shown) while an insignificant trend towards more expression of CD80 and VISTA was noted upon anti-PD-L1 treatment, especially in combination with TNF-α. More importantly, we observed a significant upregulation of LAG-3, SIRP-α and TIM-3 when monocytes were treated with recombinant TNF-α and/or anti-PD-L1 mAbs, especially when both were combined (**Figure 5A**). To validate these findings *in vivo*, we evaluated the expression levels of CD80, VISTA, LAG-3, SIRP-α and TIM-3 on freshly isolated Ly6C^+^ monocytes, derived from healthy, Ctrl or anti-PD-L1 mAb treated tumor-bearing mice. We noticed that the expression levels of all checkpoints under investigation, except LAG-3, increased on monocytes derived from tumor-bearing lungs compared to healthy controls. What’s more, anti-PD-L1 treatment fortified these elevations for CD80, VISTA, LAG-3 and TIM-3 (**Figure 5B**).

### Monocytes Play a Key Role in the CTL-Stimulating Potential of ICB Combination Therapy

**Figure 6:**
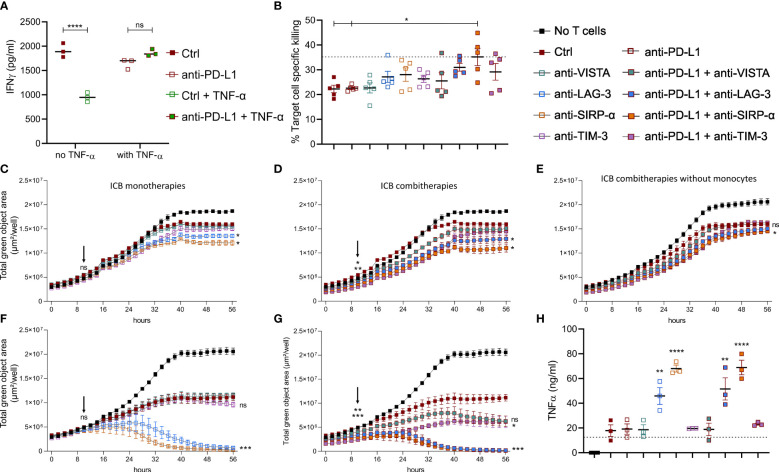
Anti-PD-L1 therapy depends on monocytes to stimulate CTLs in combination with alternative ICBs. **(A)** Dot plot depicting IFN-γ concentrations in supernatants from splenocytes 72 hrs after their incubation with 10 µg/ml Ctrl or anti-PD-L1 mAbs, with or without 1 µg/ml recombinant TNF-α (n=3). **(B–H)** depict results from LLC specific OT-I killing assays, in the presence of Ly6C^+^ monocytes sorted from lung tumor-bearing mice, no monocytes, or bone marrow derived monocytes resp. Moreover, the following conditions were compared: no monocytes + Ctrl mAb (dashed line in panels B and H), anti-PD-L1, -VISTA, -LAG-3, -SIRP-α, or -TIM-3 monotherapy **(C, F)** or their combination with anti-PD-L1 mAb **(D, E, G)**. **(B)** Percentages of cell-specific killing 3 days after experiment onset (n=1, 5 mpc). **(C–G)** Follow-up of target cell confluency *via* total green object area (µm²/well) during the respective LLC-specific OT-I killing assays over a time range of 56hrs. Images were obtained *via* Incucyte live cell imaging and statistical significances (on time points 10 and 56hrs) were calculated using a two-way ANOVA followed by a Dunnett’s multiple comparison test compared to the Ctrl mAb condition. **(H)** Dot plot depicting TNF-α concentrations in supernatants from the LLC-specific OT-I killing assays shown in **(F, G)** at the 56hrs time point (n=3). Asterisks indicate significant differences between the marked conditions and the Ctrl mAb-treated condition. In panels **(A, B, H)** a one-way ANOVA with Tukey’s multiple comparison test was used to test for statistical significance. ns, non-significant; * for p < 0.05; ** for p < 0.01; and **** for p < 0.0001.

To understand the impact of TNF-α on anti-PD-L1 mAb treated CTLs, we evaluated the immune stimulatory impact of this treatment on freshly isolated splenocytes. Notably, anti-PD-L1 mAbs induced a significant drop of IFN-γ secretion (**Figure 6A**), in line with our previous *in vivo* finding that MHC-II^low^ TAMs display a decreased gene set enrichment for IFN-γ response upon anti-PD-L1 therapy (**Supplementary Figure 5B**). Yet, when prestimulated splenocytes were treated with anti-PD-L1 mAbs and TNF-α, their IFN-γ secretion profile remained unaffected by ICB, suggesting that splenocyte activation is improved by the combination of anti-PD-L1 mAbs and TNF-α.

These data suggest that anti-PD-L1-mediated TNF-α upregulation aids the stimulation of T cells directly and indirectly *via* upregulation of CD80 on monocytes, but that this stimulatory effect is most likely counterbalanced by the upregulation of VISTA, LAG-3, SIRP-α and TIM-3.

To investigate the latter, we first performed *in vitro* LLC-eGFP/OVA target cell-specific killing by OT-I cells with or without the addition of Ly6C^+^ tumor-infiltrated monocytes, anti-PD-L1, -VISTA, -LAG-3, -SIRP-α or -TIM-3 mAbs or their combination with anti-PD-L1 (**Figures 6B-D**). When cell-specific percentages were calculated 3 days later, two important findings were observed: (1) killing dropped significantly in the presence of the Ly6C^+^ monocytes and (2) only the combination of anti-PD-L1 with anti-SIRP-α mAb could fully revert the immunosuppressive impact of these Ly6C^+^ monocytes (**Figure 6B**). Moreover, we evaluated the presence of TNF-α and IFN-γ in the supernatant of these killing assays (**Supplementary Figures 6A, B**). The respective cytokine profiles demonstrated (1) that presence of *in vivo* sorted Ly6C^+^ monocytes increased the level of TNF-α and IFN-γ in all conditions, (2) a significant rise in TNF-α when anti-PD-L1 was combined with anti-LAG-3 mAb compared to anti-LAG-3 monotherapy and (3) significant rise in IFN-γ when anti-PD-L1 was combined with anti-VISTA, anti-LAG-3 or anti-TIM-3 mAbs. By monitoring the decrease of eGFP signal (LLC) from the same killing assays, we could additionally demonstrate that also anti-LAG-3 and anti-SIRP-α monotherapies enhanced OT-I-mediated killing 3 days after their addition (**Figure 6C**). Interestingly, the addition of anti-PD-L1 mAb not only fortified but also accelerated the CTL stimulatory effect of anti-LAG-3 and - SIRP-α mAbs as a significant difference with anti-PD-L1 monotherapy was already shown 10hrs post-OT-I addition. In contrast, none of the ICB-mediated CTL-stimulating effects could be observed in the absence of monocytes, stressing the importance of monocytes for ICB mono and combi therapy effectiveness (**Figure 6E**).

Notably, very similar killing profiles were found when killing assays were repeated in the presence of *in vitro* generated bone marrow-derived monocytes (**Figures 6F–H**). As the latter showed more pronounced effects, the remarkable CTL-stimulating potential of anti-LAG-3 and anti-SIRP-α as mono- (**Figure 6F**) and combi- (**Figure 6G**) therapies was consolidated accompanied by significantly increased TNF-α secretion (**Figure 6H**). The latter killing assays further revealed that anti-VISTA and anti-TIM-3 mAbs also hold stimulatory effects when combined with anti-PD-L1 mAb on OT-I-mediated killing (**Figure 6G**) and IFN-γ secretion (**Supplementary Figure 5C**).

## Discussion

Sobering objective response rates of NSCLC patients to anti-PD-L1 therapy, stress the need for rationalized combinations to enhance therapeutic efficacy. In search for druggable targets to ameliorate ICB effectiveness, we investigated if the PD-(L)1^+^ myeloid cell compartment hampers effective anti-PD-L1 therapy in a murine ICB-unresponsive squamous NSCLC model.

By monitoring the abundance of 11 different lung-infiltrating myeloid subsets during tumor progression, a specific increase in MHC-II^high^ and MHC-II^low^ TAMs, cDC2s, Ly6C^+^, Ly6C^-^ and Tie2^+^ monocytes was shown. Moreover Ly6C^+^ monocytes in blood and CX3CR1^+^ monocytes in bone marrow showed a similar rise together with the serological level of CCL7. In line with clinical studies, the predominance of Ly6C^+^ monocytes in blood samples of NSCLC patients has been described and linked to an attenuated anti-cancer response (35, 36). Also, monocyte mobilization from bone marrow was previously reported to be managed by CCL7 (MCP-3) (37, 38) and recently found to be specifically upregulated in immature monocytes and neutrophils derived from NSCLC patient tumor parenchyma compared to adjacent non-neoplastic lung tissue (39). Previously Movahedi et al. found that Ly6C^high^ monocytes represent progenitors for TAMs in a subcutaneous LLC model (40), arguing that the rise in lung TAMs is likely preceded by an accompanying rise in peripheral Ly6C^+^ monocytes. Overall, these data suggest that the orthotopic LLC model represents a relevant squamous NSCLC model.

When LLC-bearing mice were treated with anti-PD-L1 therapy, flow cytometric analysis over time showed that the local and systemic rise in inflammatory and residential monocytes was completely abrogated. Notably, when we subjected bone marrow-derived myeloid cells to anti-PD-L1 treatment, we also observed a significant decrease in the fraction of Ly6C^+^ monocytes, suggestive of a direct, yet undetermined effect of anti-PD-L1 therapy on monocytes. These findings are further in line with a clinical case report in which it was shown that a stage III NSCLC patient undergoing durvalumab treatment (anti-PD-L1 mAb) showed a reduced number of circulating CD14^+^ and CD124^+^ myeloid cells (22).

In the assumption that most TAMs originated from Ly6C^+^ monocytes, it was not surprising that anti-PD-L1-treated mice also showed a significant decrease in their fraction of MHC-II^low^ (but not MHC-II^high^) TAMs. Notably, while 26% ± 6,7 of MHC-II^high^ TAMs were PD-L1^+^ in 3-week-old LLC tumors, this percentage was about twice as high (60% ± 21,5) for MHC-II^low^ TAMs, making the latter more vulnerable for anti-PD-L1 mAb-mediated antibody dependent cellular cytotoxicity. Notably, a previous *in vitro* study demonstrated the induction of spontaneous macrophage proliferation upon *in vitro* PD-L1 blockade (41). While this contrasts our observed decrease in MHC-II^low^ TAMs, it might offer an additional rationale for the finding that the MHC-II^high^ TAM fraction was not altered upon anti-PD-L1 therapy in the LLC model.

Functional assessment of the sorted TAMs and monocytes from anti-PD-L1-treated mice showed only insignificant trends towards a more immunostimulatory phenotype. This could partly explain the lack of ICB effectiveness. Especially as effectiveness of anti-PD-L1 therapy was previously correlated to the presence of IFN-γ-secreting activated T-cells with favorable and significant TAM remodeling (19, 42, 43). Moreover, Boutsikou et al. recently reported that increased serological levels of IFN-γ and TNF-α next to IL-1β, IL-2, IL-4, IL-5, IL-6, IL-8, IL-10 and IL-12, resulted in better responses with longer survival upon anti-PD-1 therapy in NSCLC patients (29). However, we found that serological IFN-γ levels were significantly decreased in LLC-bearing animals compared to healthy controls, irrespective of anti-PD-L1 therapy. Moreover, sorted MHC-II^low^ TAMs from anti-PD-L1-treated mice were characterized by a significantly decreased expression of IFN-γ-responsive genes, while *in vitro* treated splenocytes with anti-PD-L1 treatment reduced their capacity to secrete IFN-γ.

Recently, various inflammation-associated genes have been reported to be rapidly upregulated in blood CD14^+^ monocytes, derived from anti-PD-L1-treated NSCLC patients (23). Accordingly, we found that the lung tumor-derived monocytes sorted from ICB-treated LLC-bearing mice upregulated a gene set that was linked to the inflammation-associated cytokine TNF-α. Interestingly, this corresponded to increased expression of TNF-α by Ly6C^+^ tumor-residing monocytes with a concomitant rise of serological TNF-α levels upon anti-PD-L1-treatment *in vivo*. *In vitro*, this effect seemed to be directly caused by the presence of anti-PD-L1 mAb, as we also observed increased TNF-α secretion in the supernatants of anti-PD-L1 treated bone marrow-derived monocytes. When Lin et al. previously treated mice with anti-PD-L1 therapy, they also showed an increase in TNF-α but, in contrast to our model, also of IFN-γ, which resulted in a therapeutic benefit in the MC38 colon carcinoma model (6). Notably, Hartley et al. showed that TNF-α administration could increase the expression level of PD-L1 in bone marrow-derived macrophages and monocytes *in vitro (* 41). In contrast, it was recently shown that TNF-α-secreting TAMs found in subcutaneously LLC-engrafted mice as well as in NSCLC patients, were characterized by reduced PD-L1 expression and that their clodronate-mediated depletion led to a significant increase in PD-L1 expression in aerobic cancer cells, a higher infiltration of T cells and improved response to anti-PD-L1 therapy (44). In line with the negative impact of TNF-α-secreting TAMs, TNF-α has also been suggested to upregulate the alternative checkpoint molecule TIM-3 on lymphocytes *in vitro* and trigger activation-induced cell death of tumor-infiltrating CD8^+^ T cells in murine melanoma (33, 45). These findings rationalized at least two studies in which co-blockade of TNF-α and PD-1 was evaluated. While both studies demonstrated significant improvement of ICB efficacy in melanoma with reduced ICB-installed colitis (33, 34), we were unable to show any added benefit of TNF-α and PD-L1 co-blockade. To understand why TNF-α blockade did not ameliorate ICB effectiveness, we investigated the immunological impact of anti-PD-L1 mAb on Ly6C^+^ monocytes in the presence or absence of TNF-α. The presence of TNF-α made anti-PD-L1 mAb-treated bone marrow-derived monocytes express more CD80 (immune-stimulatory or suppressive when bound to CD28 or CTLA-4 expressed by T cells resp.) next to the alternative immunosuppressive checkpoint molecules VISTA, LAG-3, SIRP-α and TIM-3. A finding that was confirmed for each of these checkpoints in Ly6C^+^ tumor-infiltrated monocytes isolated from anti-PD-L1-treated mice, in which we previously demonstrated elevated levels of serological TNF-α. Moreover, the presence of monocytes markedly decreased the capacity of OT-I CTLs to kill LLC cells *in vitro*. Although these findings suggest the anti-PD-L1 mAb-mediated installation of immunosuppressive monocytes, their presence in the *in vitro* CTL-mediated LLC killing assays also significantly elevated the levels of IFN-γ and TNF-α, characteristic for an immunostimulatory profile. Additionally, TNF-α obliterated the anti-PD-L1 mAb-mediated decrease of IFN-γ secretion by *ex vivo* treated splenocytes. These data suggest that TNF-α plays a monocyte-associated Janus-faced role in the effectiveness of anti-PD-L1 therapy.

To investigate if we could push this see-saw profile to increased antitumor immunity upon anti-PD-L1 therapy, we finally tested the impact of VISTA, LAG-3, SIRP-α and TIM-3 co-blockade in the presence of isolated lung tumor-infiltrated monocytes. Especially co-blockade of LAG-3 or SIRP-α and PD-L1, significantly enhanced OT-I-mediated LLC killing *in vitro*, accompanied by elevated levels of TNF-α and IFN-γ. An effect that was abrogated in the absence of monocytes, pointing out their crucial role in the CTL-stimulating effect of PD-L1-LAG-3 or -SIRP-α co-blockade. In the presence of bone-marrow derived monocytes, all CTL-stimulating effects of the investigated ICB combinations were more pronounced revealing that also the blockade of VISTA and TIM-3 could ameliorate the CTL-stimulating capacity of anti-PD-L1 mAbs. Strikingly, these combinations were only able to elevate the levels of IFN-γ and not TNF-α, again proving the importance of TNF-α and monocytes to boost CTL-mediated killing upon ICB combinations and providing an explanation for the ineffectiveness of TNF-α inhibition to increase anti-PD-L1 mAb effectiveness.

Today, numerous studies have described upregulation of alternative checkpoint molecules as important culprit for anti-PD-(L)1 therapy (46). In accordance with our study, VISTA, LAG-3, and TIM-3 have been demonstrated to increase in the NSCLC tumor microenvironment rationalizing their potential as targets for NSCLC immunotherapy (47–50). Especially the initial results from the ongoing phase II study TACTI-002 on the combination of pembrolizumab with soluble LAG-3 protein showing overall response rates of 47%, are very promising (51). While most studies revealed the suppressive role of VISTA, LAG-3, and TIM-3 role in the context of T cell exhaustion (52), we uncover their suppressive role on lung tumor-infiltrated monocytes as well. Moreover, we demonstrate their upregulation of ‘don’t eat me signal’ SIRP-α too, previously shown to hamper phagocytosis upon ligation to CD47 on lung tumor cells (53–56).

In conclusion, this study divulges a Janus-faced role for monocytes upon anti-PD-L1 therapy. Hence, their biomarker potential should be considered to delineate more suited ICB combinations that increase the mediocre response rates observed in NSCLC patients today.

## Data Availability Statement

The datasets presented in this study can be found in online repositories. The names of the repository/repositories and accession number(s) can be found below: https://www.ncbi.nlm.nih.gov/geo/, GSE165517.

## Ethics Statement

All animal experiments were reviewed and approved by the Ethical Committee for Animal Experiments of the Vrije Universiteit Brussel with ECD #: 18-214-1, 18-281-9 and 20-214-14.

## Author Contributions

KDR: designing research study, conducting experiments, acquiring data, analyzing data and writing the manuscript HL: conducting experiments, acquiring data, analyzing data and manuscript proof-reading EP: conducting experiments, acquiring data, analyzing data and manuscript proof-reading MIZ: conducting experiments, acquiring data, analyzing data and manuscript proof-reading RMA: conducting experiments, acquiring data and manuscript proof-reading SV: analyzing gene expression profiling data and manuscript proof-reading MVB: support with 3D whole tissue imaging procedure and manuscript proof-reading YDV: conducting experiments and manuscript proof-reading QL: conducting experiments and manuscript proof-reading ER: conducting experiments and manuscript proof-reading WDM: conducting experiments and manuscript proof-reading LDB: conducting experiments and manuscript proof-reading TE: conducting experiments and manuscript proof-reading IP: management of the ACAM imaging core, support with 3D whole tissue imaging procedure and manuscript proof-reading J-PT.: management of the ACAM imaging core, support with 3D whole tissue imaging procedure and manuscript proof-reading DE: obtaining funding and manuscript proof-reading MK: management of the small animal imaging core facility and manuscript proof-reading KB: obtaining funding, designing research studies, analyzing data and writing the manuscript CG: obtaining funding, designing research study, conducting experiments, acquiring data, analyzing data and writing the manuscript. All authors contributed to the article and approved the submitted version.

## Funding

This research was performed with the financial support of the Research Foundation-Flanders (FWO-V, grant 1515718N, FWO-SBO, grant S004317N and FWO Junior Research, grant G041721N), Wetenschappelijk Fonds Willy Gepts of the UZ Brussel, and Kom op tegen Kanker, the Flemish Cancer Society. RMA, YDV and TE received an FWO-SB fellowship. LDB received an FWO-V fellowship. MK is senior clinical investigator at FWO-V. MIZ was funded by a PhD scholarship from Universidad Pública de Navarra, Pamplona (Spain) and DE is funded by Gobierno de Navarra (project BMED 050-2019), ISCIII (FEDER-PI17/02119, FEDER-PI20/00010) and AECC (PROYE16001ESCO). CG is funded by the Research Council of the Vrije Universiteit Brussel (OZR).

## Conflict of Interest

MK, KB, and QL have a granted patent on “Human PD-L1 binding immunoglobulins” (WO2019166622A1). MK has patents on the use of nanobodies for imaging and therapy. MK received research funding from Precirix. MK has ownership in AbScint which leverages nanobody imaging tracers into clinical application.

The remaining authors declare that the research was conducted in the absence of any commercial or financial relationships that could be construed as a potential conflict of interest.

## Publisher’s Note

All claims expressed in this article are solely those of the authors and do not necessarily represent those of their affiliated organizations, or those of the publisher, the editors and the reviewers. Any product that may be evaluated in this article, or claim that may be made by its manufacturer, is not guaranteed or endorsed by the publisher.
