# Supplementary material for: TNF-α-Secreting Lung Tumor-Infiltrated Monocytes Play a Pivotal Role During Anti-PD-L1 Immunotherapy
Source: Front Immunol. 2022 Apr 14;13:811867. doi: 10.3389/fimmu.2022.811867 (PMC9046849; doi:10.3389/fimmu.2022.811867)
Supplement: Supplementary file 1 [file DataSheet_1.docx]

**Supplementary Figure legends**

**Supplementary Figure 1: Lack of PD-L1 ICB efficacy in the orthotopic LLC model**. (A) The graph shows the murine body weights followed over the course of 21 days as a measure of tumor progression (n=3, 5 mpc). (B) Number of tumor foci formed and (C) mean tumor foci volume (µm³) measured via lightsheet microscopy in 3-week-old lungs treated with Ctrl mAb or anti-PD-L1 mAb (mpc = 4). Statistical significance was evaluated with an unpaired t-test. (D) Gating strategy to define the fraction of viable PD-L1^+^ cells within lung-derived single cell suspensions. (E) Left panel shows representative dot plot of flow cytometric PD-L1 analysis on LLC cells (grey) versus LLC cells exposed to IFN-γ for 24 hours (black). Right panel summarizes the findings from 2 independent experiments with 6 technical replicates. Statistical analysis was performed using an unpaired two tailed t-test (t=16.29, df=2).

**Supplementary Figure 2: Expression of PD-L1 on tumor-infiltrating lymphocytes and LLC cells.** (A) Optimized gating strategy for CD19^+^ B lymphocytes, CD3^+^ and CD8^+^ or CD4^+^ T lymphocytes within lung-derived single-cell suspensions from healthy and LLC-bearing mice. (B) The graph shows the percentage of CD4^+^ T-cells within the CD3^+^ population and (C) their expression of CD25 and PD-1. (D) Percentage of CD19^+^ B lymphocytes within the CD45^+^ immune cell population and (E) their expression of PD-1 (n=3, 1-3 mpc). A two-way ANOVA with Tukey’s multiple comparisons test was performed to determine statistical significance.

**Supplementary Figure 3: Myeloid cell composition and PD-L1 and PD-1 positivity in healthy versus LLC-engrafted lungs.** The lungs of healthy and LLC-Fluc tumor-bearing mice (Ctrl condition, Fig. 1A) were isolated at weeks 1, 2 and 3 of tumor development to measure PD-L1 and PD-1 expression in flow cytometry. (A) Gating strategy to distinguish different myeloid subsets from whole lung-derived cell suspension. Viable CD45^+^ (DAPI^-^) immune cells were selected. Mast cells and Tie2^+^ monocytes were defined as CD117^+^ and FCεR1a^+^, or CD11b^+^ and Tie2^+^, resp. Neutrophils were identified as Ly6G^+^ and CD11b^+^. All CD45^+^ and Ly6G^-^ cells were further analyzed for expression of CD11b and CD11c. Eosinophils were defined as CD11c^-^, CD11b^+^ and SiglecF^+^, while alveolar macrophages (AMs) were CD11c^high^ and SiglecF^high^. The MHC-II^high^, CD11c^+^ and SiglecF^-^ fraction are DCs in which conventional type-1 DCs (cDC1) and cDC2s are CD103^+^ and CD11b^-^ and CD103^-^ and CD11b^+^, resp. To define monocytes and non-AMs, we gated out the CD11c^high^ population (representing AMs and DCs) from the CD11b^+^ and Ly6G^-^ fraction (orange frame). Macrophages were defined as F4/80^+^ and further subdivided in an MHC-II^low^ and MHC-II^high^ fraction. Monocytic populations are defined by the presence of CD11b, however, absence of MHC-II. Residential monocytes were further selected on their low expression of CD11c, while the inflammatory monocytes were characterized by Ly6C. (B) Graphs indicating flow cytometrical changes in the abundance of neutrophils, eosinophils, mast cells, cDC1 and AMs within the CD45^+^ immune cell fraction from healthy or LLC-bearing lung tissue over the course of 3 weeks (upper row), and their expression of PD-L1 (middle row) and PD-1 (lower row) (n=3, 1-3 mpc). A two-way ANOVA with Tukey’s multiple comparisons statistical test was performed.

**Supplementary Figure 4: Composition and functional evaluation of different myeloid cell subsets, derived from LLC-engrafted lung tissue either treated with Ctrl or anti-PD-L1 mAb.** Mice were treated with Ctrl or anti-PD-L1 mAb on days 4, 7, 10 and 13 after LLC-Fluc injection. The graphs show the percentage of neutrophils, eosinophils, mast cells, Tie2^+^ monocytes, AMs, MHC-II^high^ macrophages (M1), cDC1 and cDC2 DCs within the CD45^+^ immune cell fraction of the lungs on week 1, 2 and 3 after tumor cell inoculation (n=3, 1-3 mpc). A two-way ANOVA with Tukey’s multiple comparisons statistical test was performed.

**Supplementary Figure 5: Effect of anti-PD-L1 and/or anti-TNF-α mAb on tumor-residing myeloid cells and tumor growth.** (A) Volcano plot of differentially expressed genes found in sorted inflammatory monocytes from anti-PD-L1 mAb compared to IC mAb-treated lungs. Adjusted p-value cut-off < 0.1 and Log2 fold change >1 and <-1. (B) Gene set enrichment analysis ranking genes against MSigDB Hallmark gene set collection for MHC-II^low^ macrophages with NES, NOM p-value and FDR q-value denoted on the graphs. On the right, the top 15 gene set members on the rank ordered list are depicted (n=1, mpc=3-4). (C-D) Mean fluorescence intensities of TNF-α obtained via flow cytometry on MHC-II^high^ (C) or ^low^ (D) macrophages isolated from healthy or LLC-bearing mice mice, treated with Ctrl or anti-PD-L1 mAb (n=1, 4-5 mpc). (E) Number of tumor foci formed and (F) mean tumor foci volume (µm³) measured via lightsheet microscopy in tumor-bearing lungs from mice treated with anti-TNF-α and/or anti-PD-L1 mAbs (mpc = 4). Statistical significance in panels C-F was determined with a one-way ANOVA followed by a Tukey’s multiple comparisons test.

**Supplementary Figure 6: TNF-α and IFN-γ secretion after target cell specific killing upon mono- or combined ICB therapy.** Cytokine concentrations were measured in supernatants after 3 days of LLC target cell specific killing by OT-I cells, supplemented with the respective mono or combination ICB therapies as depicted in the figure legend (n=1, 4 mpc). Dashed lines represent the average concentration found in the condition that lacked monocytes and was supplemented with Ctrl mAb. (A) TNF-α and (B) IFN-γ concentrations in the killing assays with *ex vivo* lung tumor sorted Ly6C^+^ monocytes (n=1, mpc=3). (C) IFN-γ concentrations in the killing assays with bone marrow-derived monocytes (n=3). A one-way ANOVA with Tukey’s multiple comparison test was used to test for statistical significance in all panels. Asterisks in panel C indicate significant differences between the marked conditions and the Ctrl treated condition.

**Supplementary Table legends**

**Supplementary Table 1:** List of all recombinant antibodies with fluorescent conjugates used for flow cytometry.

**Supplementary Table 2:** Observed concentrations of chemokines and cytokines measured via a multiplex assay on serum of healthy or LLC-engrafted mice, as mean ± SD. Statistics was performed using a one-way ANOVA followed by a Tukey’s Multiple Comparison test (df=2) (n=2, mpc=4-8). Adjusted p-values and F-values are represented in the last two columns.

**Supplementary Table 3:** Observed concentrations of chemokines and cytokines measured via a multiplex assay on serum LLC-engrafted mice either treated with Ctrl or anti-PD-L1 mAb, as mean ± SD. Statistics was performed using a one-way ANOVA followed by a Tukey’s Multiple Comparison test (df=2) (n=2, mpc=4-8). Adjusted p-values and F-values are represented in the last two columns.

**Supplementary methods
Lentiviral vector production and transduction**
Plasmids The REV, GAG, VSV.G and pCCLsin_hPGK_eGFP-WPRE transferplasmids were kind gifts from Prof. Brian Brown (Mount Sinai Icahn School of Medicine, NY). After removal of the eGFP sequence from the latter transferplasmid through a BamHI/SalI restriction digestion, a Katushka encoding gBlock with BamHI and SalI overhangs (obtained from IDT) was ligated to obtain the pCCLsin_hPGK_Katushka-WPRE transferplasmid. The transferplasmids pHR’trip_CMV_huIi80tOVA-IRES-tNGFR SIN was previously described(1).
Virus stock preparations For generation of all 3^rd^ generation lentiviral vectors (LVs), HEK293T-cells were plated at 15×1e6 cells per 175 cm^2^. These were transfected the following day using polyethyleneimine (Polysciences, Eppelheim, Germany) with 6,25; 12,5; 9 and 37,5 μg of the REV, GAG, VSV.G envelope, and transgene encoding plasmid, respectively. LV-containing supernatant was collected on days 2 and 3 and 1000x concentrated by ultracentrifugation as previously described (1,2).

**Generation of target and non-target LLC cell lines**
For generation of the murine eGFP and OVA^+^ LLC target cell line, 1e5 wild type LLC were first transduced with LVs encoding eGFP and LVs encoding OVA and truncated nerve growth factor receptor (tNGFR) (each at multiplicity of infection of 10). One week later, eGFP and tNGFR double positive cells were sorted using an BD FACSAria™III BD Biosciences to obtain the eGFP/OVA-LLC line. The Katushka (Kat)^+^ non-target LLC line was generated by transduction with LV-Kat and subsequent sort to obtain the Kat-LLC line.

1. Breckpot K, Dullaers M, Bonehill A, Meirvenne S van, Heirman C, Greef C de, Bruggen P van der, Thielemans K. Lentivirally transduced dendritic cells as a tool for cancer immunotherapy. *The Journal of Gene Medicine* (2003) 5:654–667. doi:10.1002/JGM.400

2. Liechtenstein T, Perez-Janices N, Blanco-Luquin I, Goyvaerts C, Schwarze J, Dufait I, Lanna A, Ridder M de, Guerrero-Setas D, Breckpot K, et al. Anti-melanoma vaccines engineered to simultaneously modulate cytokine priming and silence PD-L1 characterized using ex vivo myeloid-derived suppressor cells as a readout of therapeutic efficacy. *OncoImmunology* (2014) 3:e945378. doi:10.4161/21624011.2014.945378
