# Supplementary material for: TNF-α-Secreting Lung Tumor-Infiltrated Monocytes Play a Pivotal Role During Anti-PD-L1 Immunotherapy
Source: Front Immunol. 2022 Apr 14;13:811867. doi: 10.3389/fimmu.2022.811867 (PMC9046849; doi:10.3389/fimmu.2022.811867)
Supplement: Supplementary file 2 [file Presentation_1.pptx]

## Slide 1
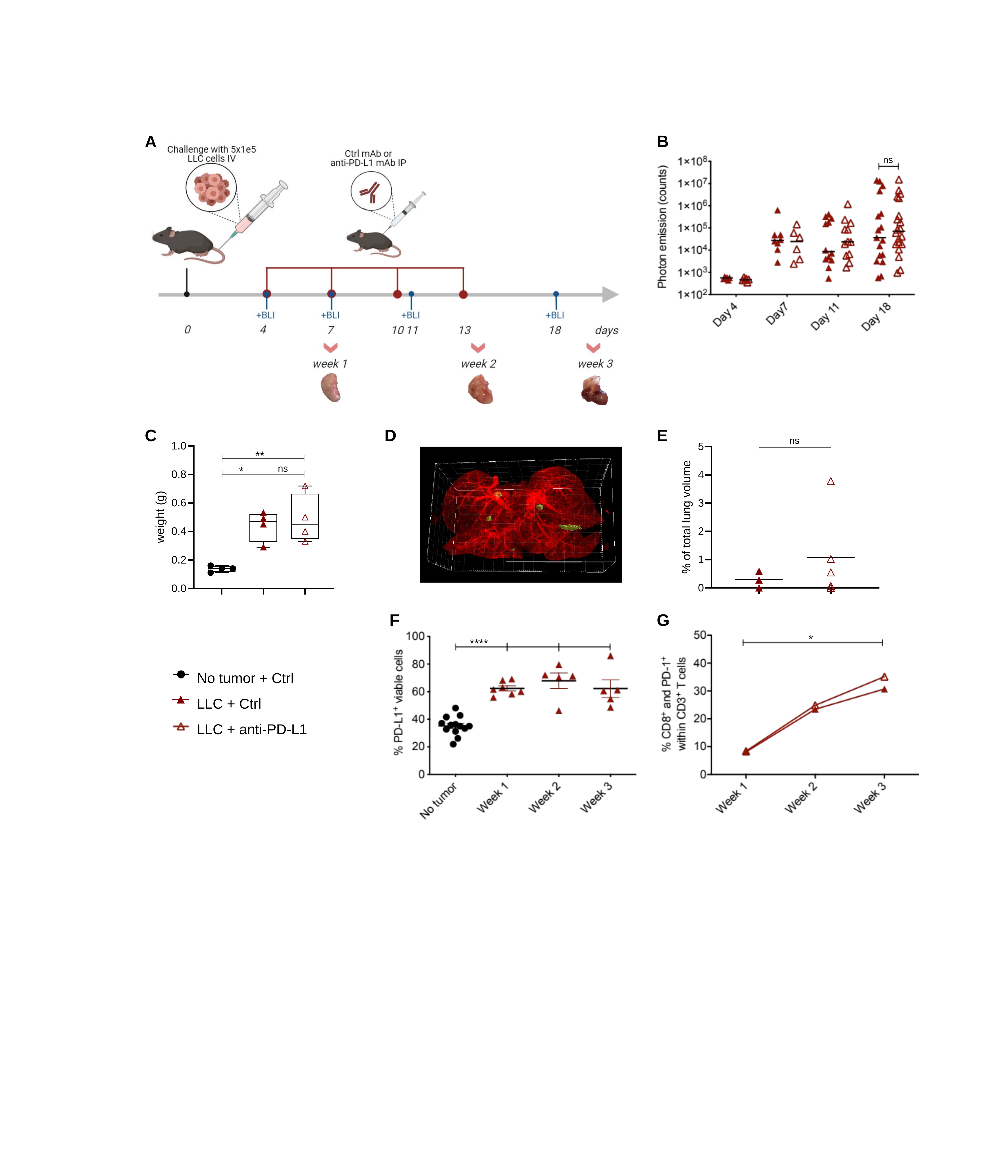

B
A
ns
C
D
E
ns
**
ns
*
F
G
*
****
No tumor + Ctrl
LLC + Ctrl
LLC + anti-PD-L1

## Slide 2
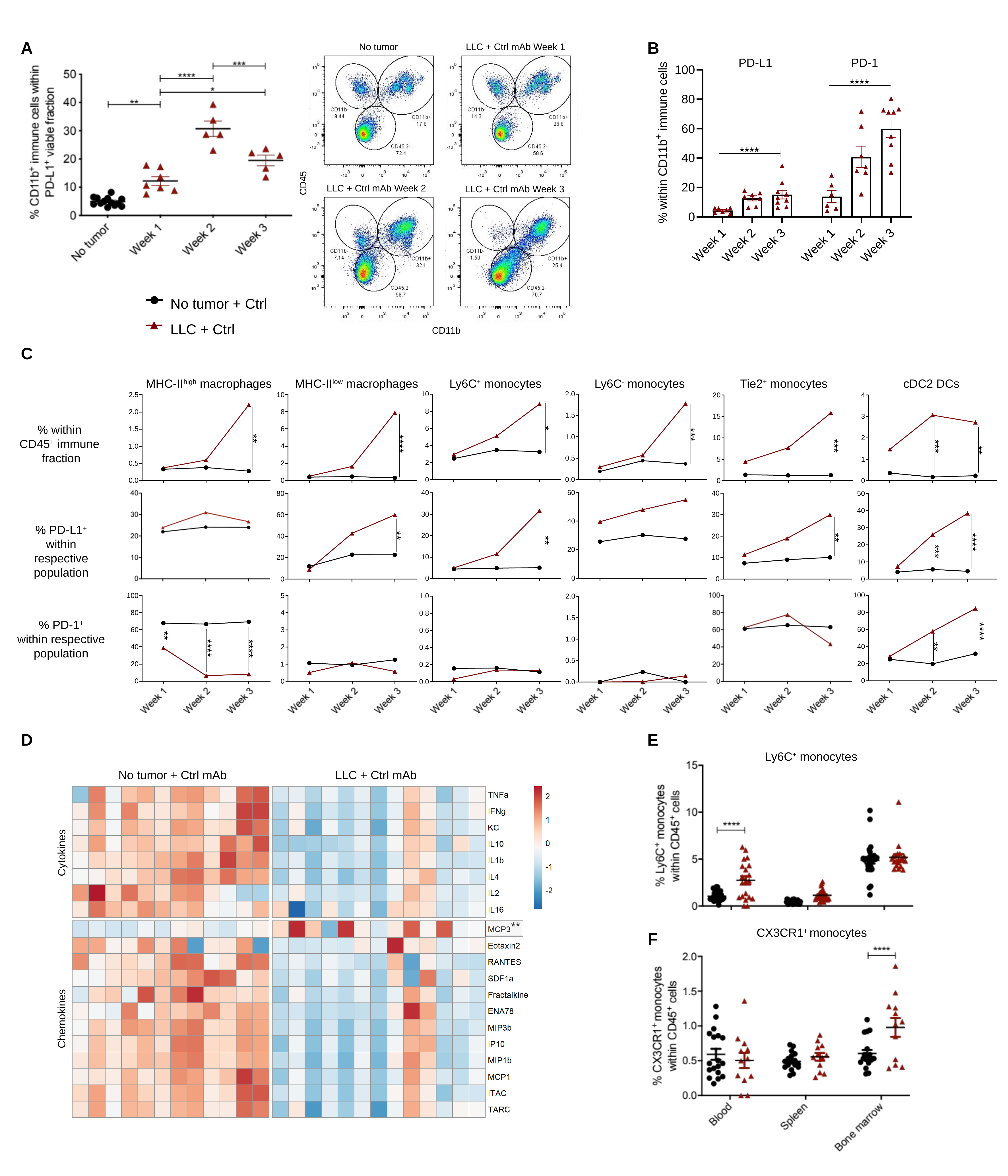

A
B
No tumor
LLC + Ctrl mAb Week 1
CD45
LLC + Ctrl mAb Week 2
LLC + Ctrl mAb Week 3
CD11b
-
-
-
-
PD-L1
PD-1
No tumor + Ctrl
LLC + Ctrl
C
MHC-IIhigh macrophages
MHC-IIlow macrophages
Ly6C+ monocytes
Ly6C- monocytes
Tie2+ monocytes
cDC2 DCs
% within
CD45+ immune
fraction
% PD-L1+
within respective population
% PD-1+
within respective population
*
***
**
****
***
***
**
**
**
**
****
***
****
**
****
****
**
D
E
Ly6C+ monocytes
LLC + Ctrl mAb
No tumor + Ctrl mAb
Cytokines
**
CX3CR1+ monocytes
F
Chemokines

## Slide 3
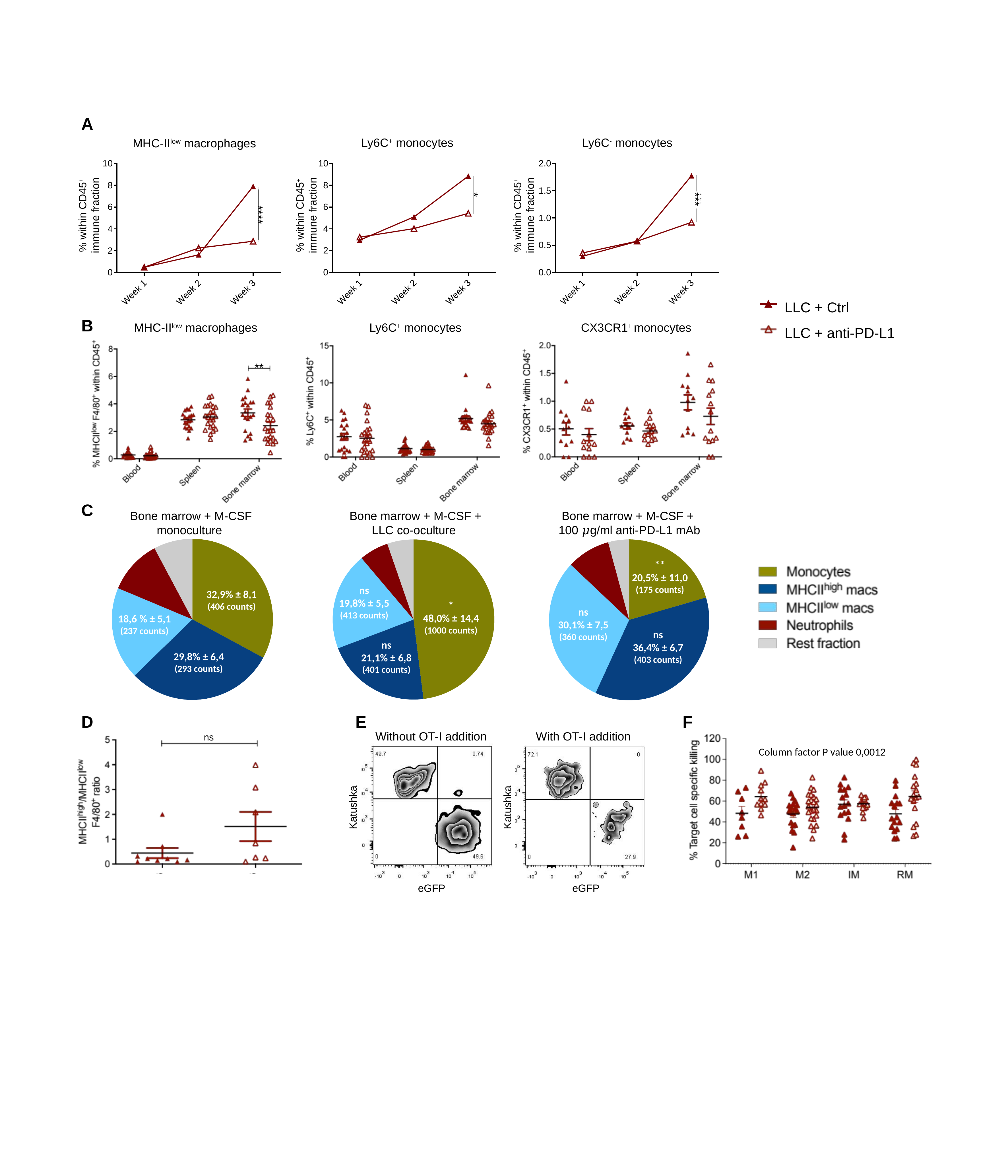

A
Ly6C+ monocytes
Ly6C- monocytes
MHC-IIlow macrophages
***
*
% within CD45+ immune fraction
% within CD45+ immune fraction
% within CD45+ immune fraction
****
LLC + Ctrl
LLC + anti-PD-L1
B
MHC-IIlow macrophages
Ly6C+ monocytes
CX3CR1+ monocytes
**
C
Bone marrow + M-CSF monoculture
32,9% ± 8,1
(406 counts)
18,6 % ± 5,1
(237 counts)
29,8% ± 6,4
(293 counts)
Bone marrow + M-CSF + LLC co-oculture
ns
19,8% ± 5,5
(413 counts)
*
48,0% ± 14,4
(1000 counts)
ns
21,1% ± 6,8
(401 counts)
Bone marrow + M-CSF +
100 𝜇g/ml anti-PD-L1 mAb
**
20,5% ± 11,0
(175 counts)
ns
30,1% ± 7,5
(360 counts)
ns
36,4% ± 6,7
(403 counts)
D
E
F
Without OT-I addition
With OT-I addition
Katushka
Katushka
eGFP
eGFP
ns
Column factor P value 0,0012

## Slide 4
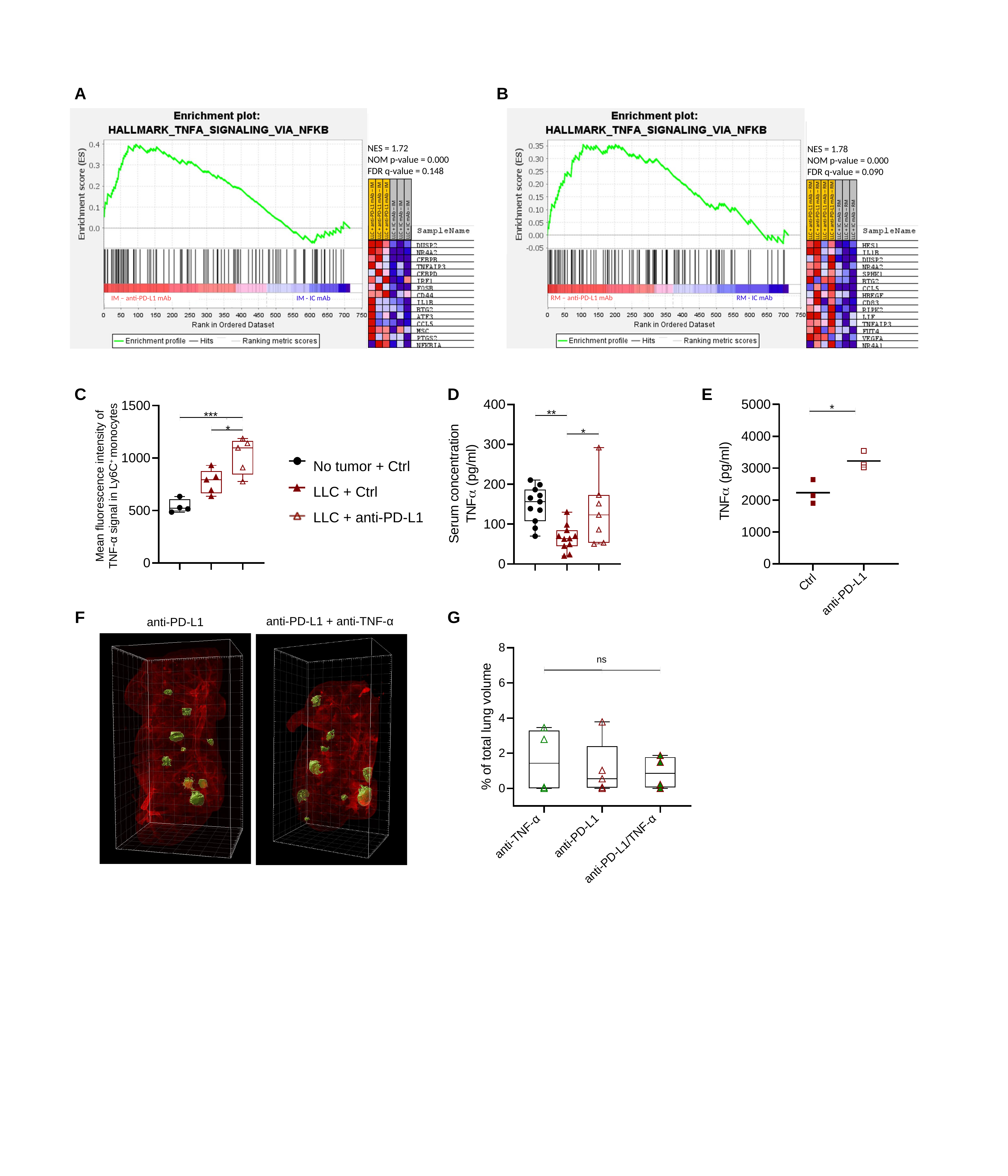

A
B
NES = 1.72
NOM p-value = 0.000
FDR q-value = 0.148
IM – anti-PD-L1 mAb
IM - IC mAb
NES = 1.78
NOM p-value = 0.000
FDR q-value = 0.090
RM – anti-PD-L1 mAb
RM - IC mAb
C
D
E
*
**
***
*
*
No tumor + Ctrl
LLC + Ctrl
LLC + anti-PD-L1
Mean fluorescence intensity of TNF-α signal in Ly6C+ monocytes
Ctrl
anti-PD-L1
F
G
anti-PD-L1 + anti-TNF-α
anti-PD-L1
ns
anti-PD-L1
anti-TNF-α
anti-PD-L1/TNF-α

## Slide 5
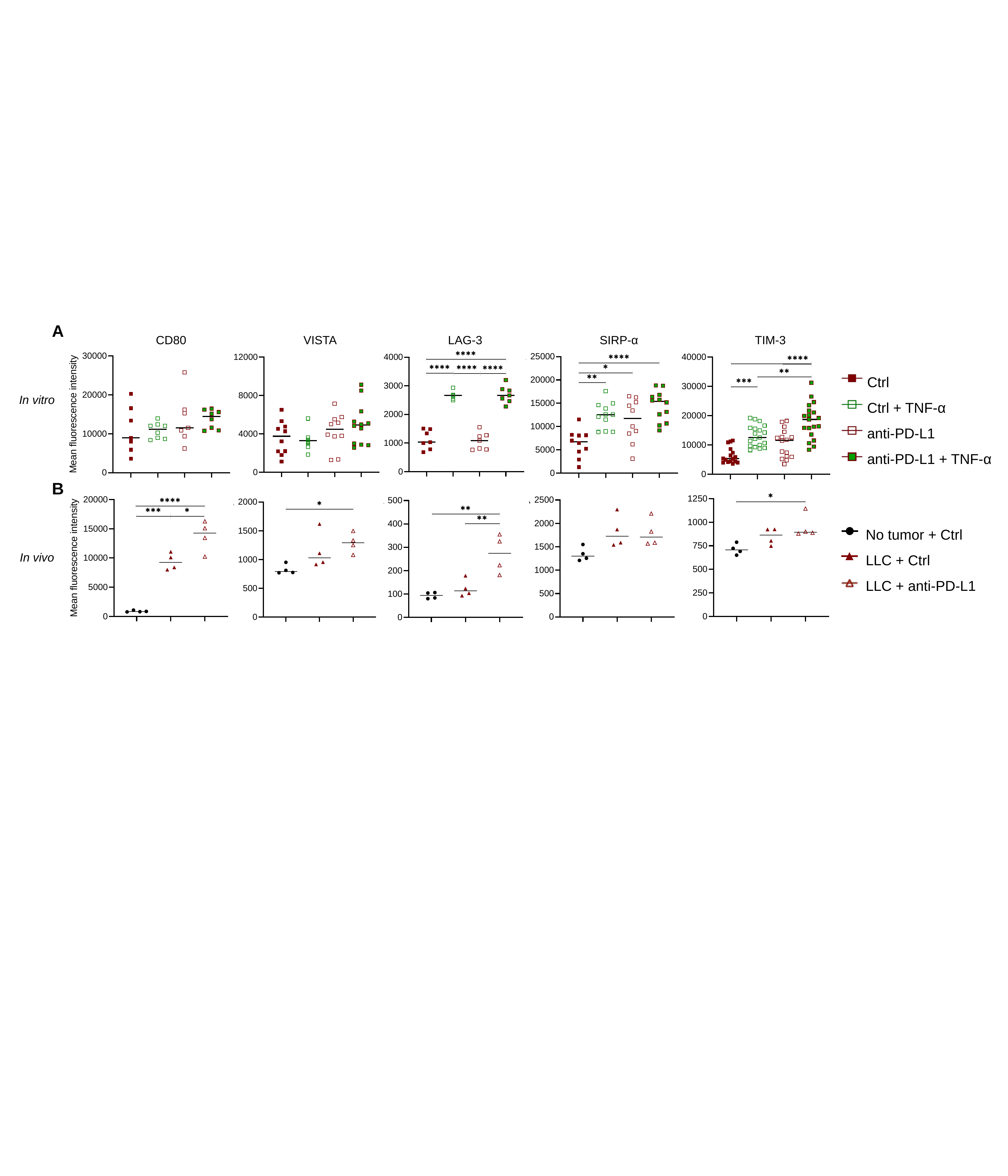

A
CD80
VISTA
LAG-3
SIRP-α
TIM-3
Ctrl
Ctrl + TNF-α
anti-PD-L1
anti-PD-L1 + TNF-α
In vitro
B
No tumor + Ctrl
LLC + Ctrl
LLC + anti-PD-L1
In vivo

## Slide 6
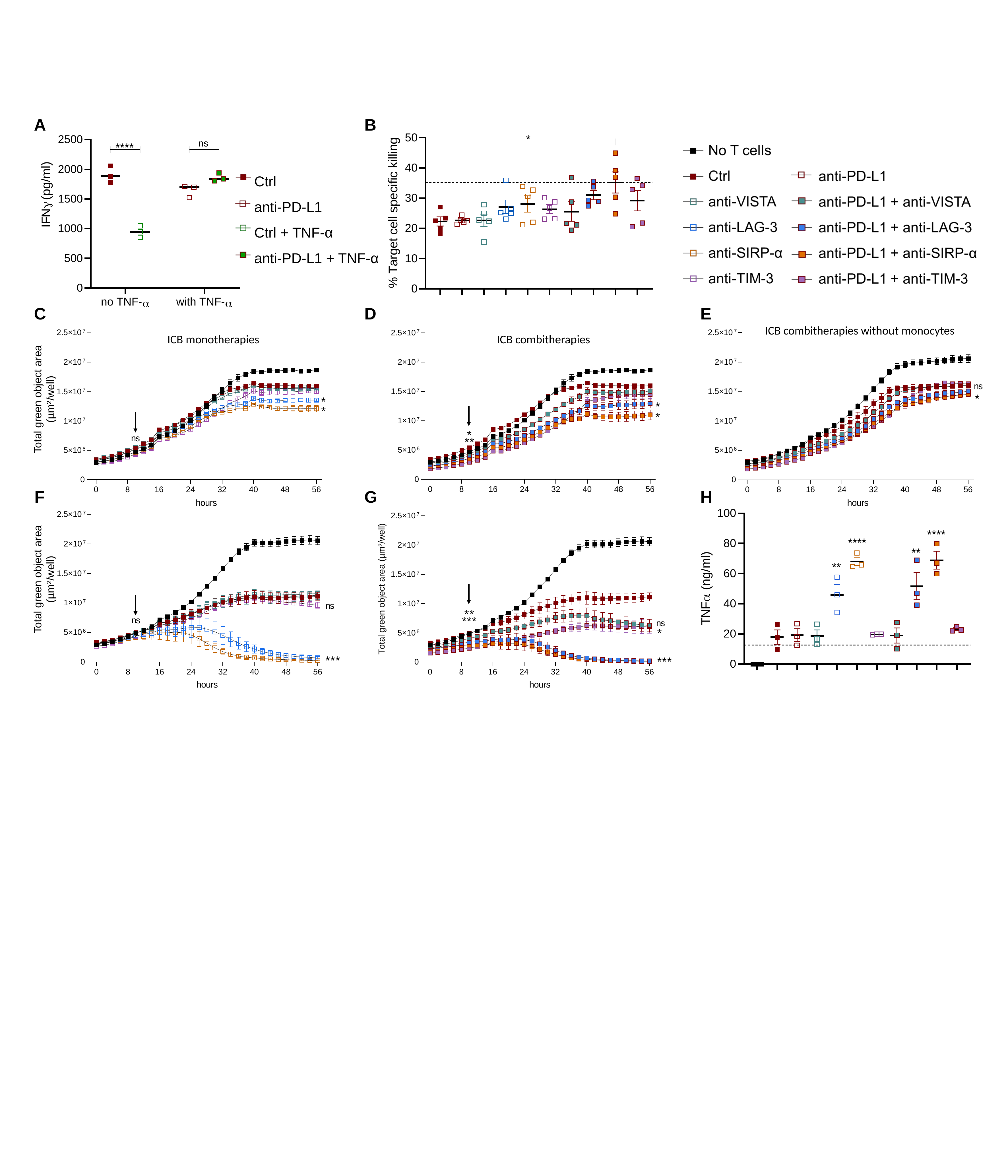

A
B
*
ns
****
Ctrl
anti-PD-L1
Ctrl + TNF-α
anti-PD-L1 + TNF-α
C
D
E
ICB combitherapies without monocytes
ICB monotherapies
ICB combitherapies
Total green object area (µm²/well)
F
G
H
****
****
**
**
Total green object area (µm²/well)
